# Preliminary analysis of self-reported quality health indicators of patients on opioid agonist therapy at specialty and primary care clinics in Ukraine: A randomized control trial

**DOI:** 10.1371/journal.pgph.0000344

**Published:** 2022-11-02

**Authors:** Oleksandra Pashchenko, Daniel J. Bromberg, Kostyantyn Dumchev, Katherine LaMonaca, Iryna Pykalo, Myroslava Filippovych, Denise Esserman, Maxim Polonsky, Samy J. Galvez de Leon, Olga Morozova, Sergii Dvoriak, Frederick L. Altice

**Affiliations:** 1 Geisel School of Medicine at Dartmouth, Hanover, NH, United States of America; 2 Department of Social and Behavioral Sciences, Yale School of Public Health, Yale University, New Haven, CT, United States of America; 3 Yale Center for Interdisciplinary Research on AIDS, Yale University, New Haven, CT, United States of America; 4 Ukrainian Institute on Public Health Policy, Kyiv, Ukraine; 5 Yale School of Medicine, Yale University, New Haven, CT, United States of America; 6 European Institute on Public Health Policy, Kyiv, Ukraine; 7 Department of Biostatistics, Yale School of Public Health, New Haven, Connecticut, United States of America; 8 Keck Graduate Institute, Claremont, CA, United States of America; 9 Department of Public Health Sciences, Biological Sciences Division, University of Chicago, Chicago, IL, United States of America; University of Milano–Bicocca: Universita degli Studi di Milano-Bicocca, ITALY

## Abstract

International agencies recommend integrating addiction treatment into primary care for people who inject drugs (PWID) with opioid use disorder (OUD). Empirical data supporting integration that incorporates comprehensive health outcomes, however, are not known. For this randomized controlled trial in Ukraine, adult PWID with OUD were randomized to receive opioid agonist therapy (OAT) in specialty addiction treatment clinics (SATC) or to primary care clinics (PCCs). For those randomized to PCC, they were subsequently allocated to PCCs where clinicians received pay-for-performance (P4P) incentives (PCC with P4P) or not (PCC without P4P). Participating cities had one of each of the three intervention sites to control for geographic variation. Ongoing tele-education specialty training (OAT, HIV, tuberculosis) was provided to all PCCs. While the primary outcome for the parent trial focuses on patient medical record data, this preliminary analysis focuses on assessment of self-reported achievement of nationally recommended quality health indicators (QHIs) which is summed as a composite QHI score. Secondary outcomes included specialty and primary care QHI subscores. This study occurred from 01/20/2018-11/1/2020 with 818 of 990 randomized participants having complete self-reported data for analysis. Relative to SATC (treatment as usual), the mean composite QHI score was 12.7 (95% CI: 10.1–15.3; p<0.001) percentage points higher at PCCs; similar and significantly higher scores were observed in PCCs compared to SATCs for both primary care (PCC vs SATC: 18.4 [95% CI: 14.8–22.0; p<0.001] and specialty (PCC vs SATC: 5.9 [95% CI: 2.6–9.2; p<0.001] QHI scores. Additionally, the mean composite QHI score was 4.6 (95% CI: 2.0–7.2; p<0.001) points higher in participants with long term (>3 months) experience with OAT compared to participants newly initiating OAT. In summary, PWID with OUD receive greater primary care and specialty healthcare services when receiving OAT at PCCs supported by tele-education relative to treatment as usual provided in SATCs.

**Clinical trial registration:** This trial was registered at clinicaltrials.gov and can be found using the following registration number: NCT04927091.

## Introduction

Opioid agonist therapies (OAT) are the most effective treatment for opioid use disorder (OUD) [1] and their scale-up is crucial to mitigate the rapidly evolving HIV epidemic in Eastern Europe, which is largely concentrated among people who inject drugs (PWID) with OUD [2, 3]. Modeling studies suggest that OAT scale-up is necessary for controlling the HIV epidemic and reducing death in Ukraine and similar Eastern European and Central Asian countries [4–6] where OAT and other HIV program coverage is sub-optimal [2, 3]. Mathematical modeling studies suggest that the most cost effective and feasible way to scale-up OAT in Ukraine is through expansion from specialty addiction treatment clinics (SATC) to primary care clinics (PCC) due to a limited number of specialists and the need for geographic diversity [7]. By year-end 2019, OAT was prescribed for only 4.5% of PWID with OUD in Ukraine, well below internationally recommended targets [8], in part due to the low number of addiction treatment specialists to support OAT scale-up [7].

PWID with OUD have high levels of co-morbid conditions that contribute to increased mortality [9]. On average, these individuals die 10–25 years earlier than the overall population, mostly from conditions that can be screened for and treated in PCCs [10, 11]. Until a recent pilot demonstration project [12], OAT was unavailable in PCCs in Ukraine and limited to SATCs that commonly provide co-located HIV and/or tuberculosis (TB) services [13], but no primary care. SAT clinics are fewer in number, often have inconvenient locations, and limited hours of operation [12, 13]. Integrating services into a single location such as PCCs can increase access to clinical care, reduce the long waiting lists generally found at specialty locations, and increase uptake of recommended healthcare services [12–16].

In November 2017, the national legislation governing OAT delivery in Ukraine was changed to allow OAT to be provided in any healthcare setting, including PCCs, and not restricted to addiction treatment specialists, thus forming the basis for integrating OAT into PCCs [7, 12]. Simultaneously, the National Health Service (NHS) of Ukraine, which pays clinics based on the number of patients and type of care provided to patients, was created as part of the first stage of a national healthcare reform to strengthen primary care.

Although there is increasing evidence and recommendations supporting the expansion of OAT into PCCs globally, previous studies have identified that lack of training, stigma and negative attitudes towards OAT prevent non-specialists from providing care to PWID [12, 13, 17, 18]. To address this problem, tele-education platforms that connect non-specialists with specialists have been adapted from Project Extension for Community Healthcare Outcomes (ECHO) to provide education, training, support and consultation via tele-health technology [19]. Project ECHO has becoming increasingly popular internationally [20] and has the potential to positively impact the global epidemics of drug injection and HIV. This method of training, however, had not yet been deployed in Ukraine.

Healthcare providers in low- and middle-income countries (LMIC) like Ukraine are mostly underpaid and seldom receive incentives for their performance [17, 21]. Providing pay-for-performance (P4P) incentives to physicians is recommended by the World Health Organization and the Institute of Medicine to improve quality of care [15, 22, 23]. To direct P4P incentives and assess the level of care patients receive, clinical management, government organizations and researchers have increasingly used quality health indicators (QHIs) [24]. Wiessing et al. [25] emphasize using standardized QHIs for PWIDs to better understand the healthcare they are receiving; however, few empirical studies have made use of standardized QHIs for PWID [13, 26].

To address the need to expand OAT nationally and overcome complex healthcare needs of PWID with OUD in Ukraine, we conducted a multi-site, prospective trial to compare QHIs for PWID with OUD randomized to receive OAT in SATC or PCCs. Participants randomized to receive OAT at PCC then selected (blinded to status) a clinic that had been randomized to P4P or no P4P for physicians throughout regions of Ukraine.

## Methods

### Ethics statement

This study was reviewed and approved by the institutional review boards at Yale University and Ukrainian Institute on Public Health Policy. All participants provided written informed consent with a release of medical information for chart surveys, which are part of the parent trial.

### Study design

**Fig 1:**
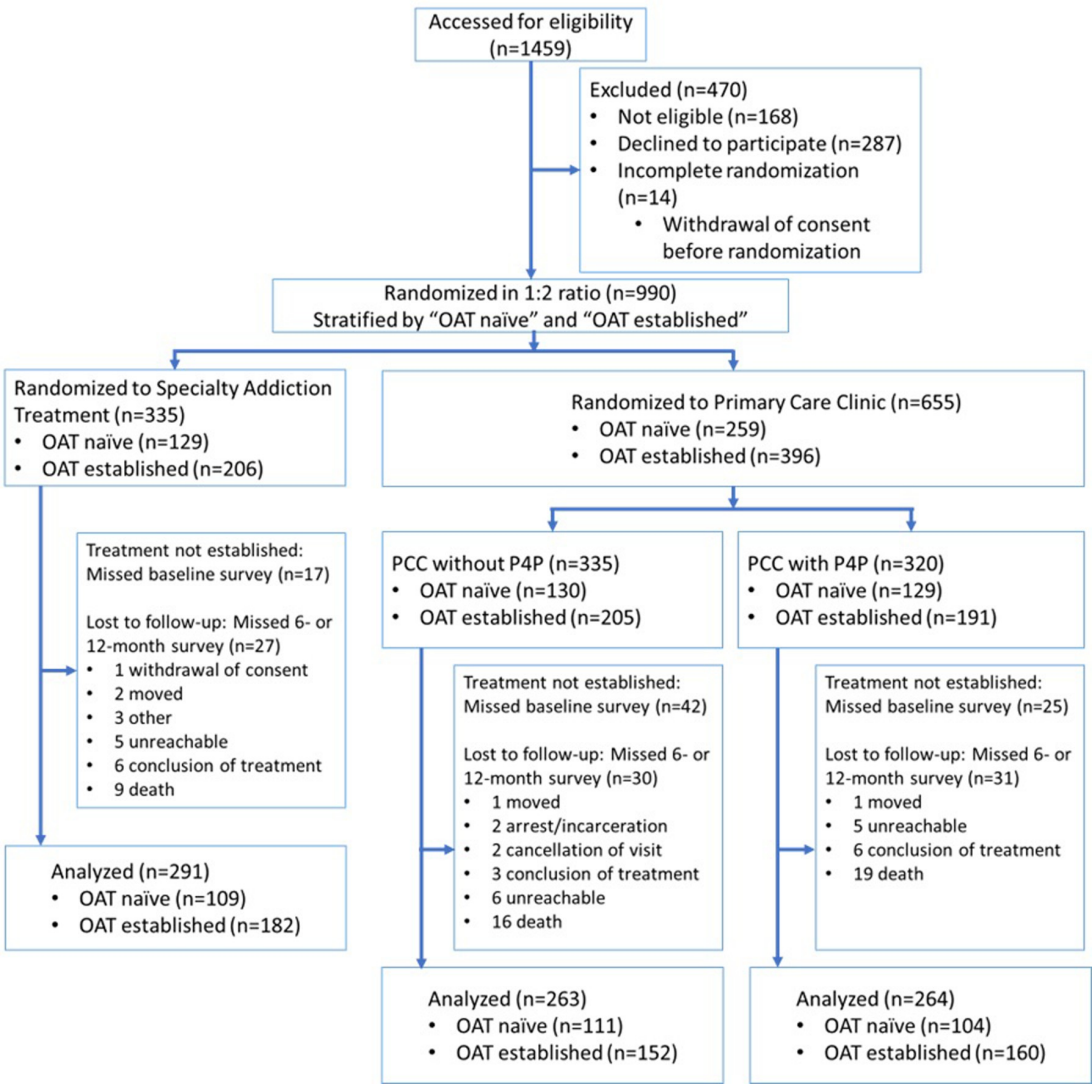
Participant disposition in the three study arms: Flow diagram of participants enrolled, randomized, and excluded from the study. Abbreviations: OAT: opioid agonist therapy, PCC without P4P: primary care clinic without pay-for-performance; PCC with P4P: primary care clinic with pay-for-performance.

**Fig 2:**
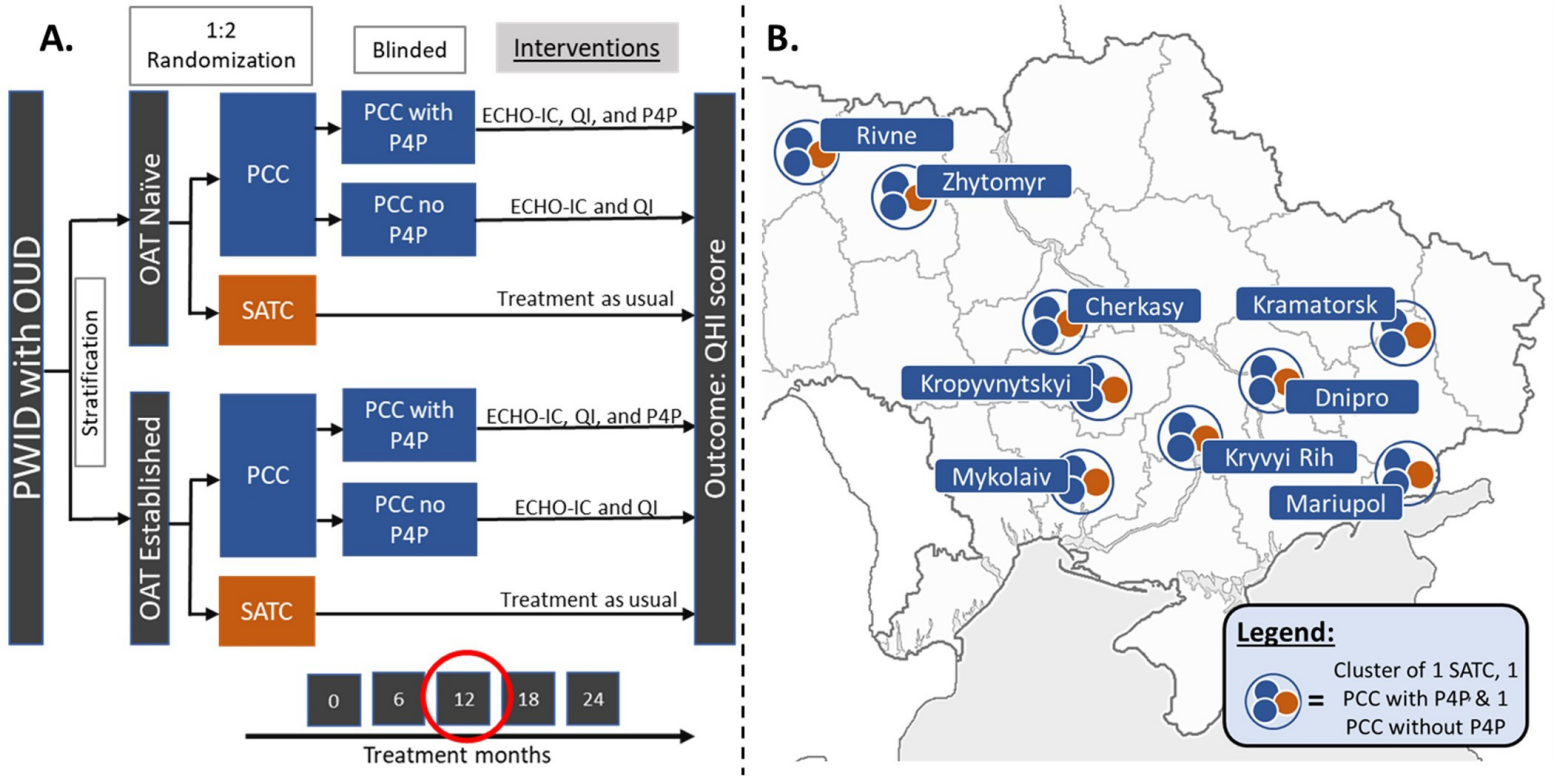
Study design and distribution of regions. A). The outline of the study design with interim analysis at 12 months. B) Distribution of regions included in analysis with each region containing a cluster of one SATC, one PCC with P4P, and one PCC without P4P. The map was obtained from via Wikimedia Commons (https://commons.wikimedia.org/wiki/File:Map_of_Ukraine_blank.png) as a public domain image with several changes made to the image: colored markers indicating rough approximations or city locations, colored legend, image was cropped and colors were changed to black and white. Abbreviations: ECHO-IC: Extension for Community Healthcare Outcomes-Integrated Care; OAT: opioid substitiute therapy; OUD: opioid use disorder; PCC without P4P: primary care clinic without pay-for-performance; PCC with P4P: primary care clinic with pay-for-performance; PWID: people who inject drugs; QHI: quality health indicator; QI: quality improvement; SATC: specialty addiction treatment clinic.

A randomized controlled trial is now underway to compare outcomes between OAT delivered in SATC versus delivery in PCC with P4P or without P4P for physicians. The study design is outlined in **Figs 1 and 2A**. This interim analysis was conducted as part of the data safety and monitoring plan recommendations to assess self-reported outcomes; the main outcome for the parent trial is objective QHIs obtained through chart review. The main trial involves 12 regions, but due to delays in rolling out regions in sequential phases, preliminary 12-month data are examined for only 9 regions of Ukraine: Cherkasy, Dnipro, Kramatorsk, Kropyvnytskyi, Kryvyi Rih, Mariupol, Mykolaiv, Rivne, and Zhytomyr. To account for geographical variation, participants in each region were distributed among one of three groups: SATC, PCC with P4P, and PCC without P4P as indicated in **Fig 2B**. Each of the sites aimed to enroll half of their participants from patients who were already stable on OAT (OAT established) and the other half from patients newly enrolling onto OAT (OAT naïve). Each region was phased into the trial at four start-up times to allow for adequate allocation of resources and research personnel. The study was registered with clinicaltrials.gov after enrollment began due to changes in staffing near the beginning of the study causing a delay in registration.

### Participants

People seeking care or already receiving care at SATCs were approached for the study. Eligibility criteria included age 18 years or older, meeting ICD-10 criteria for opioid dependence, interested in or already on OAT, any past injection drug use, and residing within the legal catchment district to receive primary care. Exclusion criteria included being under police investigation, planning on moving to a new area, inability to provide informed consent, and inability to understand Ukrainian or Russian fluently.

### Randomization

We utilized permuted block randomization with variable block sizes to randomize eligible participants 1:2 to SATC or PCC using a computerized randomization module in REDCap [27]. Enrolled participants were stratified based on whether they were stable on OAT (3 months or more in treatment) or newly starting OAT. Those randomized to PCCs could choose their preferred PCC (often choosing one closest to their home) but were blinded to the P4P status of the PCC. P4P status was randomly assigned to one of the two participating PCCs within a local geographic region. Research assistants oversaw enrollment activities, and the data manager oversaw participant randomization using REDCap [27] and informed the research assistants.

### Study sites and intervention

After enrollment, research assistants advised all participants to seek primary care services, irrespective of their randomization. Participants randomized to PCC then chose one of the two PCCs (PCC with P4P or PCC without P4P) in their region, blinded to the P4P status. Those randomized to SATC, the current standard of care in Ukraine, continued to receive OAT services at their respective SATC and primary care services at the one in their district, with no other intervention imposed aside from structured surveys. Those randomized to receive OAT in PCCs, irrespective of P4P incentives, received care at PCCs where the clinical staff completed two 3-day training courses. The first course focused on regulations regarding OAT and basic training on how to manage OAT, HIV and TB. Staff were also oriented to how to effectively use ECHO-Integrated Care (ECHO-IC), which would provide more advanced and ongoing training and clinical support on management of HIV, OAT, and TB in a primary care setting. The second 3-day training course focused on quality improvement (QI) strategies. Following this, each PCC continued to participate in weekly ECHO-IC sessions that consisted of clinical didactic and case presentations along with QI strategies focused on topics surrounding care for PWID. Clinicians at PCC with P4P were also informed that they could earn a monetary monthly bonus depending on the number of QHIs completed by their patients.

### Outcomes

For this preliminary analysis we collected self-reported surveys from participants at six and twelve months about their healthcare utilization at any location in the previous six months, specifically focusing on pre-determined QHIs. The QHI survey included questions such as “Have you had a medical examination completed in the past six months?” Additional information about demographics, HIV, HCV, and TB was obtained via survey at baseline and each following interview. Technical information such as name and date of birth was collected directly by the interviewer, who was blinded to P4P status but not PCC vs SATC status, and the rest of the survey including QHI outcome data was self-administered with the interviewer in the room to answer any questions the participant had. Reasons for early withdrawal from the study were collected via phone interviews with the participant or a personal contact (friend/relative); a list of contacts was provided by each participant at the beginning of the trial to be used in case of inability to contact the participant. All data were collected and managed via RedCAP [27].

**Fig 3:**
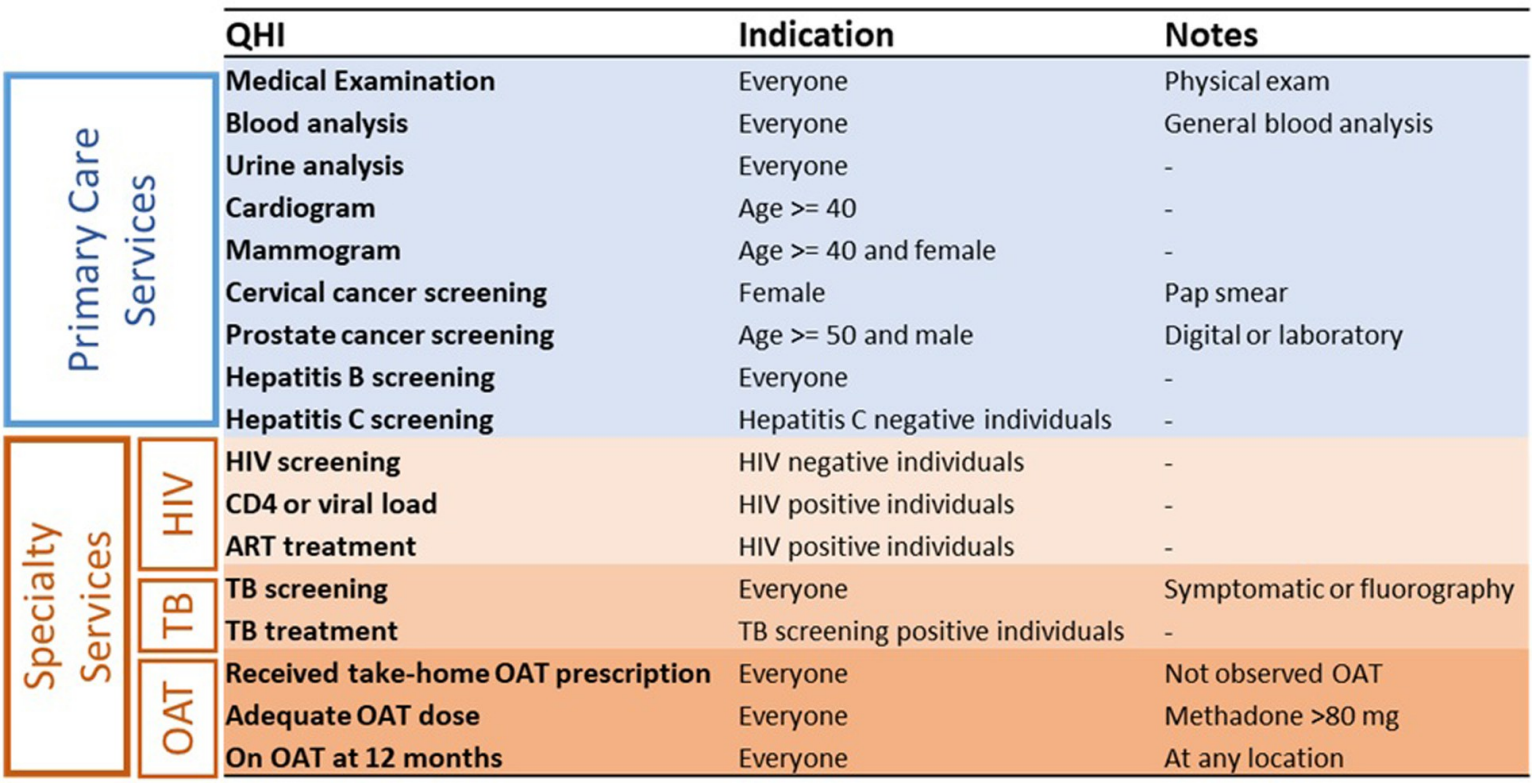
A review of primary and specialty care quality health indicators. Quality health indicators and for whom the service is recommended. Abbreviations: ART: antiretroviral treatment; HIV: human immunodeficiency virus; OAT: opioid agonist therapy; QHI: Quality health indicator; TB: tuberculosis.

QHIs were developed using Ukraine Ministry of Health guidelines and a group of national and international experts who finalized the QHIs using the Delphi method; all QHIs used in this analysis are in accordance with the current national clinical guidelines in Ukraine [28, 29]. Some services are recommended to be completed bi-annually, annually, or less frequently; however, for each study participant, one completed QHI in the twelve month period of the study satisfied the requirements to be included in the composite QHI score used for analysis. The final selected QHIs for the study are summarized in **Fig 3**. Some QHIs were only indicated for patients based on their age, sex (e.g., mammography), TB or HIV status (**Fig 3**). The completion of recommended services for individuals was summarized as a QHI score (QHI score = [number of services completed/number of services recommended)] x 100%). The composite QHI score (mean QHI score including all services) was the primary outcome for this analysis. Secondary outcomes included QHI subgroups, specifically for primary care (mean QHI score including only primary care services) and specialty treatment QHIs score (mean QHI score including only OAT, HIV and TB services).

The primary outcome of the parent trial is verified QHIs from patient clinical records at the end of 24 months; the primary outcome for this preliminary analysis is self-reported at the end of 12-months. These preliminary self-reported findings were presented to an independent Data Safety and Monitoring Board (DSMB) as part of an interim analysis that included two US and one Ukrainian expert. These findings, along with the observation of no major harms observed, resulted in the DSMB to recommend study continuation since the findings did not meet futility criteria.

### Statistical analysis

Analyses were performed using STATA SE 15 [30], R v.4.0X [31], and SAS 9.4 [32]. Power calculations for the primary outcome of the parent trial, showed a sample size of 405 per group (total sample size of 1215) was needed to have 90% power at a 5% level of significance to detect a standardized effect size of 0.10 for a null of no difference between the means of the three groups versus an alternative of at least one difference at the 24-month time point. A final sample size of 1350, after inflation by 10% for potential loss to follow-up at 24 months, was deemed necessary to have 90% power with a 2.5% type I error rate (Bonferonni corrected) to detect a standardized effect size of 0.16 within each of the OAT strata (new versus experienced).

A linear mixed model was used (after verification of model assumptions) to estimate the least squares mean QHI for SATC compared to PCC treatment group, OAT status groups, and P4P groups adjusting for region as a random effect. Pairwise comparisons were used to determine mean differences between QHI scores for each group. To assess the impact of missing data, we conducted a sensitivity analysis using an extreme allocation value single imputation method. To bias against our hypothesis, patients at integrated care settings will have higher QHI than those treated at addiction specialty settings, we assigned all patients with missing outcome data in integrated care a QHI score of 0 (the lowest possible) and those in addiction specialty a QHI score of 100 (the highest possible).

## Results

Enrollment and data collection for 1459 participants occurred from January 20, 2018 to November 1, 2020 (**Fig 1**) in 9 regions of Ukraine. Of the 990 patients randomized, all were receiving (n = 602) or newly initiating methadone (n = 388). The sample size in our initial power calculation for the final endpoint was not yet reached for this preliminary analysis. Of the randomized participants, 84 did not complete the baseline survey mainly due to withdrawal of consent, and 95 were missing either 6-month or 12-month surveys with the main reasons being death, inability to reach the participant, or arrest/incarceration. Of the 84 participants that did not complete the baseline survey, there was a higher proportion of missed baseline surveys in the PCC without P4P group (n = 42) which was mainly due to one site, Kryvyi Rih, where deviation from the protocol led to early withdrawal by participants. This was remediated early in the study. Of the 95 participants that missed either 6-month or 12-month surveys, the number of participants lost to follow up did not significantly differ between the three groups. Participants with missing data due to death were more likely to have needed treatment for TB treatment or more likely to have HIV. Cause of death for several of the individuals was TB or HIV and is the likely reason for this correlation. Additionally, sensitivity analysis with extreme values (0) imputed for the QHI scores in PCC with P4P and PCC without P4P in all missing surveys did not change the conclusion of the primary analysis. Due to the results of these sensitivity analyses, participants with missing 6-month or 12-month data were omitted from the analysis. A total of 172 (17.4%) participants were omitted from the final analysis due to incomplete data (missing baseline, 6-month, or 12-month surveys).

The characteristics of the 906 participants with completed baseline information **(Table 1)** were similar between the PCC and SACT groups with participants being on average 40.2 years old, mostly male (82.3%) and 46.7% had HIV, and the distribution of participants by region is outlined in **S1 Table**. Similarly, the characteristics between individuals in the P4P compared to without P4P were similar **(S2 Table). Table 2** describes the self-reported completion rate of each individual QHI by treatment group (SATC vs PCC). The services that were completed at <20% were screening for cervical, prostate, and breast cancer (mammograms), and treatment of TB. **S3 Table** presents the completion rates within the PCC group broken down by P4P status.

**Table 1:** Baseline characteristics of study participants (N = 906).

	SATC (n = 318)	PCC (n = 588)	Total (n = 906)
**Mean age in years** (SD)	40.1 (7.9)	40.2 (7.7)	40.2 (7.7)
**Male**	260 (81.8%)	486 (82.7%)	746 (82.3%)
**Unemployed**	170 (53.5%)	265 (45.1%)	435 (48.0%)
**Income below poverty (<1630 UAH/month)**	124 (39.0%)	209 (35.6%)	333 (36.8%)
**Married or cohabitating**	109 (34.3%)	197 (33.5%)	306 (33.8%)
**Housing**			
Lives in own house/apartment	80 (25.0%)	75 (25.6%)	232 (25.6%)
Living with family	193 (60.7%)	181 (61.8%)	561 (61.9%)
Other	45 (14.2%)	37 (12.6%)	113 (12.5%)
**Support for OAT at home**			
Members at home support OAT	235 (73.9%)	403 (68.5%)	638 (70.4%)
Members at home do not support OAT	20 (6.3%)	61 (10.4%)	81 (8.9%)
Lives alone	63 (19.8%)	124 (21.1%)	187 (20.6%)
**Above Secondary Education**	197 (62.0%)	362 (61.6%)	559 (61.7%)
**HIV status**			
HIV positive	152 (47.8%)	271 (46.1%)	423 (46.7%)
HIV negative	152 (47.8%)	282 (48.0%)	434 (47.9%)
Unknown	14 (4.4%)	35 (6.0%)	49 (5.4%)
**HCV status**			
HCV positive	188 (59.1%)	343 (58.3%)	531 (58.6%)
HCV negative	73 (22.8%)	136 (23.1%)	209 (23.0%)
Unknown	57 (17.8%)	109 (18.5%)	166 (18.3%)

Abbreviations: ART: antiretroviral treatment; HCV: hepatitis C virus; HIV: human immunodeficiency virus; OAT: opioid agonist therapy; PCC without P4P: primary care clinic without pay-for-performance; PCC with P4P: primary care clinic with pay-for-performance; SAT: specialty addiction treatment; SD: standard deviation; TB: tuberculosis; UAH: Ukrainian hryvnia.

**Table 2:** Quality health indicators achieved over the first 12 months of observation, stratified by treatment allocation (N = 818).

	SATC (n = 291)		PCC (n = 527)		Total (n = 818)	
Quality Health Indicator	Indicated	% Completed	Indicated	% Completed	Indicated	% Completed
Medical exam	291	79.4%	527	83.5%	818	82.0%
Blood analysis	291	76.6%	527	91.5%	818	86.2%
Urine analysis	291	72.2%	527	87.3%	818	81.9%
Cardiogram	134	40.3%	246	76.0%	380	63.4%
Mammogram	26	0.0%	54	11.1%	80	7.5%
Cervical cancer screening	52	7.7%	91	20.9%	143	16.1%
Prostate cancer screening	32	0.0%	55	7.3%	87	4.6%
Hep B screening	291	29.6%	527	61.9%	818	50.4%
Hep C screening	115	50.4%	222	79.3%	337	69.4%
HIV screening	154	71.4%	289	90.3%	443	83.8%
CD4/viral load	137	92.0%	238	92.4%	375	92.3%
ART treatment	137	83.2%	238	86.1%	375	85.1%
TB screening	291	73.5%	527	84.3%	818	80.4%
TB treatment	46	21.7%	71	11.3%	117	15.4%
Received take-home OAT	291	38.1%	527	49.7%	818	45.6%
Adequate OAT Dose	291	64.6%	527	67.4%	818	66.4%
On OAT at 12 months	291	91.4%	527	95.3%	818	93.9%

Abbreviations: ART: antiretroviral treatment; Hep: hepatitis; OAT: opioid agonist therapy; PCC without P4P: primary care clinic without pay-for-performance; PCC with P4P: primary care clinic with pay-for-performance; QHI: quality health indicator; SATC: specialty addiction treatment clinic; TB: tuberculosis.

**Fig 4:**
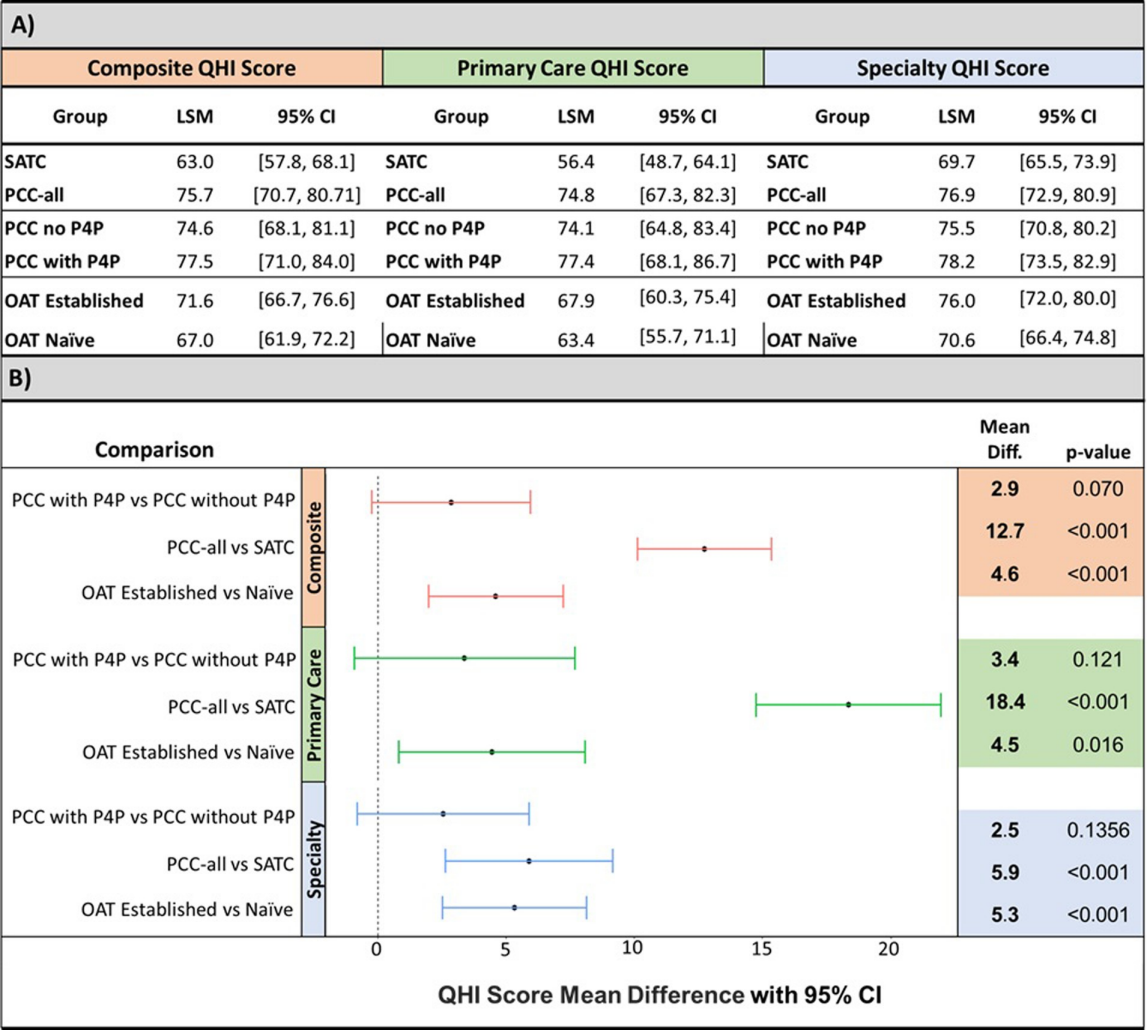
Least square means and mean differences of QHI scores between groups. (A) Least square mean estimates for each treatment group and OAT stratification. (B) QHI score mean differences for pairwise comparisons of treatment groups and OAT status. (N = 818). Abbreviations: CI: confidence interval; LSM: least mean square; OAT: opioid agonist therapy; PCC without P4P: primary care clinic without pay-for-performance; PCC with P4P: primary care clinic with pay-for-performance; QHI: quality health indicator; SATC: specialty addiction treatment clinic.

The self-reported completion of QHIs by each participant is summarized as a least square mean for each treatment group in **Fig 4A** and QHI score mean differences for pairwise comparisons shown in **Fig 4B**. The primary outcome of mean composite QHI score was 12.7 (95% CI: 10.1–15.3; p<0.001) percentage points higher at PCC compared to SATC. A similar difference is observed for the secondary analysis of the primary care QHI score where PCC was 18.4 (95% CI: 14.8–22.0; p<0.001) percentage points higher compared to SATC. A smaller yet statistically significant difference is seen for the specialty care (OAT/HIV/TB) QHI score which was on average 5.9 (95% CI: 2.6–9.2; p<0.001) percentage points higher in PCC compared to SATC. For all three QHI scores, no statistically significant differences were observed between the two PCC groups (PCC without P4P and PCC with P4P).

When comparing QHI scores for individuals who were stable on OAT for 3 months or longer at the start of the trial (OAT established) vs individuals who were newly starting OAT (OAT naïve), participants on established OAT regimens had significantly higher QHI scores in all three categories: composite, primary care, and specialty. The composite QHI score was 4.6 (95% CI: 2.0–7.2; p<0.001) percentage points higher in the group already established on OAT compared to the OAT naïve group.

## Discussion

Despite international recommendations to integrate and expand primary care for PWID, there have been few empiric evaluations of its effects on clinical outcomes, especially regarding more comprehensive health outcomes. To fill this gap, we review interim self-report data from a prospective randomized trial to assess integration of OAT into PCCs using ECHO-IC facilitation to ensure that primary care clinicians have the skills necessary to provide specialty care services. Important among these findings is that PWID who are treated with OAT at PCCs with ECHO-informed support obtained significantly more comprehensive care, measured as composite QHI scores, than those treated with OAT at SAT clinics–the current standard of care. These composite QHI score improvements achieved for patients at PCCs were driven independently by both the additional primary care services provided, measured as primary care QHIs, but also by specialty (OAT, HIV, TB) service QHIs. Importantly, the specialty care QHIs which included addiction treatment services, were at least as good in primary care as in SAT settings suggesting that primary care providers with support from ECHO-IC can manage not only the addiction specialty conditions, but potentially other infectious diseases like HIV and TB for which providers received additional support. Of note, however, low levels of TB indicators in all treatment groups suggest that there may be additional barriers to providing this service that are not addressed with ECHO-IC. One potential explanation is that the primary care doctors do not have routine access to tools and funding to better manage TB [33], a specialty that is tightly regulated and has a fee service in the new National Health System of Ukraine that provides independent payments for this service.

Improved comprehensive care for people with substance use disorders are important because previous research has shown that these individuals have a higher mortality, largely from chronic diseases that can be screened for and treated in primary care settings [9–11]. Though this study does not make the link between healthcare delivered and mortality, the access to primary care for PWID suggests that there might be downstream benefits from the additional screening and treatment that can be provided in primary care settings. Longer studies will be needed to examine downstream effects.

Integrated models for people with substance use disorder have previously demonstrated improvement in addiction related outcomes and infectious disease treatments [13, 34, 35], however, few studies demonstrate the effect of integration of services on primary care outcomes [26, 36–38]. One study that included primary care measures such as hypertension and sexually transmitted infections showed highly improved treatment completion when services were provided on-site compared to off-site referral [38]. Another study, however, showed that co-location of addiction and primary care services resulted in markedly lower levels of addiction severity, but did not influence the combined outcome of perceived health, functional limitations, and self-perceived severity of any physical co-morbid medical conditions [39]. While standardized QHIs have been recommended to assess quality of health [25], only one previous study utilized primary care QHIs to demonstrate improved QHI completion when OAT was prescribed by primary care providers compared to specialty providers [26, 37]. Information from prior studies along with our interim analysis seems to suggest that co-located services have several benefits, however longer term investigations may be needed to analyze the link between primary care for people with substance use disorders and disability, hospitalization or mortality. Increased engagement between patients and the healthcare system may result in other positive outcomes including increased patient satisfaction, convenience, and reduced stigma [12, 17, 40]. Consequently, integrated or even co-located services are crucial to provide health equity for PWID to bridge the gap in health service delivery.

In our preliminary results, patients newly initiating OAT compared to patients stable on OAT for longer than 3 months had significantly lower comprehensive QHI scores. This indicates that there may patient-level or provider-lever factors that may impact the care that is received by patients who are newly entering substance use treatment programs. One potential explanation is that patients that are newly being inducted onto OAT require considerable time initially to focus on addressing addiction-related issues and only when patients are stabilized, can other secondary health-related issues be addressed. In the parent study, chart review can disentangle the timing of QHIs achieved and at the end of 24 months, these indicators may achieve parity.

Surprisingly, there was no statistically significant incremental benefit observed in QHIs for patients treated in PCCs with P4P incentives, although trends were in the direction of P4P. In a setting like Ukraine where physicians earn extraordinarily low salaries, we expected significantly higher QHIs for those with incentives, relative to those without them [22]. Despite discussions with primary care clinicians before the study, the incentive structure may not have been suitable for this setting. For example, incremental increases in payment, failure to incorporate loss aversion theory into the P4P incentive structure, or payments being too infrequent could have explained its seemingly lack of benefit [41]. Though generally P4P strategies are a key recommendation in aiding the expansion of services and improved care at PCCs [15, 22], most studies show mixed outcomes [42]. Qualitative studies are needed to further explore these findings and to guide improvements in incentives for primary care physicians in Ukraine.

Despite these early findings that suggest that integrating OAT into PCCs can substantially improve the comprehensiveness of care delivered to PWID with OUD, there are several limitations. This study represents an interim analysis, and final reports should include all study sites, follow participants over a complete 24 months, and include verified data from the patient medical record. Verified data are crucial to reduce any social desirability bias and recall bias which can be present with self-reported data, although we see no reason to believe that the recall would be differential between the groups and bias our estimates, and it is important to observe outcomes over a longer time-period as there are some services for which patients may not need on annual basis. Additionally, the level of clinical significance of the QHI score reported in this manuscript is difficult to ascertain. While a 12-percentage point difference in QHI score could mean 1–2 more healthcare services obtained by a patient, those services may be TB treatment or a urine analysis which may hold different weight for the patient’s overall health. QHI scores may be more clinically meaningful if the services were weighed based on level of importance which is difficult to determine and varies on an individual basis. With these limitations in mind, it is equally important to report on these preliminary findings as they show promising beneficial impact on the health of PWID, and these results can begin guiding the expansion of OAT in resource limited settings.

Additional limitations for this preliminary analysis include: (a) The study was not powered for this preliminary look at the 12-month self-report data. Thus, in light of not having a clear definition of clinical significance and the difference between P4P groups not reaching statistical significance, it makes those differences difficult to interpret; however, differences in QHI scores between SATC and PCC were significant, indicating the strong positive effect of the intervention; (b) Although we are observing a strong effect size in the self-report data, the effect size may not be an accurate reflection of the final effect size in the full sample for the 12-month self-report data or at the final 24-month endpoint, which will be based on chart review; [43, 44] (c) While recommended QHI completion frequency is variable (semi- to bi-annually), we limited our QHI achievement to only if it had been done in the past year, potentially leading to over or under-recording of scores. The QHIs observed in this study encompass a comprehensive array of services, but the composite QHI score does not account for the variable importance of completing some QHIs compared to others; and (d), we excluded individuals without complete data and while our conclusions did not change after conducting extreme value imputation, there is potential for these results to have been biased. Nevertheless, these findings provide preliminary but important insights into understanding integration of OAT into PCCs in Ukraine.

## Supporting information

S1 Table Distribution of participants by region of Ukraine. (DOCX)

S2 Table Baseline characteristics of primary care groups stratified by pay for performance status. (N = 588). Abbreviations: ART: antiretroviral treatment; HCV: hepatitis C virus; HIV: human immunodeficiency virus; OAT: opioid agonist therapy; PCC without P4P: primary care clinic without pay-for-performance; PCC with P4P: primary care clinic with pay-for-performance; SATC: specialty addiction treatment; SD: standard deviation; TB: tuberculosis; UAH: Ukrainian hryvnia.
(DOCX)

S3 Table Quality health indicators achieved at primary care groups over 12 months, stratified by pay for performance status. (N = 527). Abbreviations: ART: antiretroviral treatment; Hep: hepatitis; OAT: opioid agonist therapy; PCC without P4P: primary care clinic without pay-for-performance; PCC with P4P: primary care clinic with pay-for-performance; QHI: quality health indicator; SATC: specialty addiction treatment clinic; TB: tuberculosis.
(DOCX)

S1 Protocol Trial protocol as written prior to commencement of the trial. (DOCX)

S1 Checklist Randomized trial checklist. (PDF)

S1 Text Inclusivity in global health questionnaire. (DOCX)
