# Supplementary material for: Preliminary analysis of self-reported quality health indicators of patients on opioid agonist therapy at specialty and primary care clinics in Ukraine: A randomized control trial
Source: PLOS Glob Public Health. 2022 Nov 2;2(11):e0000344. doi: 10.1371/journal.pgph.0000344 (PMC10021202; doi:10.1371/journal.pgph.0000344)
Supplement: S1 Table — (DOCX) [file pgph.0000344.s001.docx]

**S1 Table:** Distribution of participants by region of Ukraine

| **Region** | **Freq.** | **Percent** |
| --- | --- | --- |
| Rivne | 24 | 2.7 |
| Mariupol | 55 | 6.1 |
| Zhytomyr | 86 | 9.5 |
| Kropyvnytskyi | 101 | 11.2 |
| Dnipro | 107 | 11.8 |
| Cherkasy | 110 | 12.1 |
| Kramatorsk | 128 | 14.1 |
| Mykolaiv | 136 | 15.0 |
| Kryvyi Rih | 159 | 17.6 |
| **Total** | **906** | **100**.**0** |
