# Supplementary material for: Preliminary analysis of self-reported quality health indicators of patients on opioid agonist therapy at specialty and primary care clinics in Ukraine: A randomized control trial
Source: PLOS Glob Public Health. 2022 Nov 2;2(11):e0000344. doi: 10.1371/journal.pgph.0000344 (PMC10021202; doi:10.1371/journal.pgph.0000344)
Supplement: S2 Table — (N = 588). Abbreviations: ART: antiretroviral treatment; HCV: hepatitis C virus; HIV: human immunodeficiency virus; OAT: opioid agonist therapy; PCC without P4P: primary care clinic without pay-for-performance; PCC with P4P: primary care clinic with pay-for-performance; SATC: specialty addiction treatment; SD: standard deviation; TB: tuberculosis; UAH: Ukrainian hryvnia. (DOCX) [file pgph.0000344.s002.docx]

**S2 Table:** Baseline characteristics of primary care groups stratified by pay for performance status. (N=588). Abbreviations: ART: antiretroviral treatment; HCV: hepatitis C virus; HIV: human immunodeficiency virus; OAT: opioid agonist therapy; PCC without P4P: primary care clinic without pay-for-performance; PCC with P4P: primary care clinic with pay-for-performance; SATC: specialty addiction treatment; SD: standard deviation; TB: tuberculosis; UAH: Ukrainian hryvnia.

|  | **PCC without P4P (n=293)** | **PCC with P4P (n=295)** |
| --- | --- | --- |
| **Mean age in years** (SD) | 40.0 (7.6) | 40.5 (7.7) |
| **Male** | 241 (82.3%) | 245 (83.1%) |
| **Unemployed** | 130 (44.4%) | 135 (45.8%) |
| **Income below poverty (<1630 UAH/month)** | 96 (32.8%) | 114 (38.6%) |
| **Married or cohabitating** | 90 (30.7%) | 107 (36.3%) |
| **Housing** |  |  |
| Lives in own house/apartment | 75 (25.6%) | 77 (26.1%) |
| Living with family | 181 (61.8%) | 187 (63.4%) |
| Other | 37 (12.6%) | 31 (10.5%) |
| **Support for OAT at home** |  |  |
| Members at home support OAT | 193 (65.9%) | 210 (71.2%) |
| Members at home do not support OAT | 36 (12.3%) | 25 (8.5%) |
| Lives alone | 64 (21.8%) | 60 (20.3%) |
| **Above Secondary Education** | 189 (64.5%) | 173 (58.6%) |
| **HIV status** |  |  |
| HIV positive | 142 (48.5%) | 129 (43.7%) |
| HIV negative | 138 (47.1%) | 144 (48.8%) |
| Unknown | 13 (4.4%) | 22 (7.5%) |
| **HCV status** |  |  |
| HCV positive | 169 (57.7%) | 174 (59.0%) |
| HCV negative | 69 (23.6%) | 67 (22.7%) |
| Unknown | 55 (18.8%) | 54 (18.3%) |
