# Supplementary material for: Preliminary analysis of self-reported quality health indicators of patients on opioid agonist therapy at specialty and primary care clinics in Ukraine: A randomized control trial
Source: PLOS Glob Public Health. 2022 Nov 2;2(11):e0000344. doi: 10.1371/journal.pgph.0000344 (PMC10021202; doi:10.1371/journal.pgph.0000344)
Supplement: S3 Table — (N = 527). Abbreviations: ART: antiretroviral treatment; Hep: hepatitis; OAT: opioid agonist therapy; PCC without P4P: primary care clinic without pay-for-performance; PCC with P4P: primary care clinic with pay-for-performance; QHI: quality health indicator; SATC: specialty addiction treatment clinic; TB: tuberculosis. (DOCX) [file pgph.0000344.s003.docx]

**S3 Table:** Quality health indicators achieved at primary care groups over 12 months, stratified by pay for performance status. (N=527). Abbreviations: ART: antiretroviral treatment; Hep: hepatitis; OAT: opioid agonist therapy; PCC without P4P: primary care clinic without pay-for-performance; PCC with P4P: primary care clinic with pay-for-performance; QHI: quality health indicator; SATC: specialty addiction treatment clinic; TB: tuberculosis.

|  | **PCC without P4P (n=263)** | | **PCC with P4P (n=264)** | |
| --- | --- | --- | --- | --- |
| **Quality Health Indicator** | Indicated | % Completed | Indicated | % Completed |
| Medical exam | 263 | 81.8% | 264 | 85.2% |
| Blood analysis | 263 | 91.6% | 264 | 91.3% |
| Urine analysis | 263 | 86.3% | 264 | 88.3% |
| Cardiogram | 124 | 73.4% | 122 | 78.7% |
| Mammogram | 29 | 6.9% | 25 | 16.0% |
| Cervical cancer screening | 48 | 22.9% | 43 | 18.6% |
| Prostate cancer screening | 23 | 0.0% | 32 | 12.5% |
| Hep B screening | 263 | 57.0% | 264 | 66.7% |
| Hep C screening | 112 | 75.9% | 110 | 82.7% |
| HIV screening | 135 | 94.1% | 154 | 87.0% |
| CD4/viral load | 128 | 92.2% | 102 | 92.7% |
| ART treatment | 128 | 85.9% | 110 | 86.4% |
| TB screening | 263 | 82.9% | 264 | 85.6% |
| TB treatment | 34 | 14.7% | 37 | 8.1% |
| Received take-home OAT | 263 | 44.9% | 264 | 54.6% |
| Adequate OAT Dose | 263 | 63.5% | 264 | 71.2% |
| On OAT at 12 months | 263 | 93.5% | 264 | 97.0% |
