# Supplementary material for: Preliminary analysis of self-reported quality health indicators of patients on opioid agonist therapy at specialty and primary care clinics in Ukraine: A randomized control trial
Source: PLOS Glob Public Health. 2022 Nov 2;2(11):e0000344. doi: 10.1371/journal.pgph.0000344 (PMC10021202; doi:10.1371/journal.pgph.0000344)
Supplement: S1 Protocol — (DOCX) [file pgph.0000344.s004.docx]

**
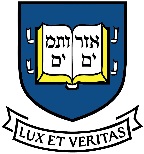
YALE UNIVERSITY**

**HUMAN INVESTIGATION COMMITTEE**

**Application to Involve Human Subjects in Biomedical Research**

**100 FR1 (2015-2)**

## Section I: Administrative Information

| **Title of Research Project:** Integrating Addiction Treatment and HIV Services into Primary Care Clinics in Ukraine | | | | | | |
| --- | --- | --- | --- | --- | --- | --- |
| **Principal Investigator:** Frederick Altice, MD | | | | **Yale Academic Appointment:** Professor | | |
| **Department:** Internal Medicine, AIDS Program | | | | | | |
| **Campus Address:** 135 College Street, Suite 323 | | | | | | |
| **Campus Phone:** 737-2883 | | **Fax:** | | | **Pager:** | **E-mail:** Frederick.altice@yale.edu |
| **Protocol Correspondent Name & Address (***if different than PI***):** Ruthanne Marcus | | | | | | |
| **Campus Phone:** 764-9958 | **Fax:** | | **E-mail:** Ruthanne.marcus@yale.edu | | | |
| **Yale Cancer Center CTO Protocol Correspondent Name & Address (***if applicable***):** | | | | | | |
| **Campus Phone:** | **Fax:** | | **E-mail:** | | | |
| **Business Manager:** | | | | | | |
| **Campus Phone :** | **Fax :** | | **E-mail** | | | |

| **Faculty Advisor:**(required if PI is a student, resident, fellow or other trainee)  **NA** | | | **Yale Academic Appointment:** | |
| --- | --- | --- | --- | --- |
| **Campus Address:** | | | | |
| **Campus Phone:** | **Fax:** | **Pager:** | | **E-mail:** |

**Investigator Interests**:

Does the principal investigator, or do any research personnel who are responsible for the design, conduct or reporting of this project or any of their family members (spouse or dependent child) have an incentive or interest, financial or otherwise, that may affect the protection of the human subjects involved in this project, the scientific objectivity of the research or its integrity? Note: The Principal Investigator (Project Director), upon consideration of the individual’s role and degree of independence in carrying out the work, will determine who is responsible for the design, conduct, or reporting of the research.

See Disclosures and Management of Personal Interests in Human Research <http://www.yale.edu/hrpp/policies/index.html#COI>

Yes X No

Do you or does anyone on the research team who is determined by you to be responsible for the design, conduct or reporting of this research have any patent (sole right to make, use or sell an invention) or copyright (exclusive rights to an original work) interests related to this research protocol?

Yes X No

If yes to either question above, list names of the investigator or responsible person:

*The Yale University Principal Investigator, all Yale University co-investigators, and all Yale University individuals who are responsible for the design, conduct or reporting of research must have a current financial disclosure form on file with the University’s Conflict of Interest Office. Yale New Haven Hospital personnel who are listed as co-investigators on a protocol with a Yale University Principal Investigator must also have a current financial disclosure form on file with the University’s Conflict of Interest Office.* If this has not been done, the individual(s) should follow this link to the COI Office Website to complete the form:  <http://www.yale.edu/coi/>

NOTE: The requirement for maintaining a current disclosure form on file with the University’s Conflict of Interest Office extends primarily to Yale University and Yale-New Haven Hospital personnel.  **Whether or not they are required to maintain a disclosure form with the University’s Conflict of Interest Office, all investigators and individuals deemed otherwise responsible by the PI who are listed on the protocol are required to disclose to the PI any interests that are specific to this protocol.**

## Section II: General Information

1. **Performing Organizations:** Identify the hospital, in-patient or outpatient facility, school or other agency that will serve as the location of the research. Choose all that apply:

**a. Internal Location[s] of the Study:**

Magnetic Resonance Research Center  Yale University PET Center

(MR-TAC)  YCCI/Church Street Research Unit (CSRU)

Yale Cancer Center/Clinical Trials Office (CTO)  YCCI/Hospital Research Unit (HRU)

Yale Cancer Center/Smilow  YCCI/Keck Laboratories

Yale-New Haven Hospital  Yale-New Haven Hospital—Saint Raphael Campus

Cancer Data Repository/Tumor Registry

Specify Other Yale Location:

**b. External Location[s]:**

APT Foundation, Inc.  Haskins Laboratories

Connecticut Mental Health Center  John B. Pierce Laboratory, Inc.

Clinical Neuroscience Research Unit (CNRU) Veterans Affairs Hospital, West Haven

Other Locations, Specify:  International Research Site

(Specify location(s)): Ukrainian Institute on Public Health Policy, Kiev, Ukraine

**c. Additional Required Documents *(check all that apply)*:**  N/A

*YCCI-Scientific and Safety Committee (YCCI-SSC) Approval Date:

*Pediatric Protocol Review Committee (PPRC) Approval Date:

*YCC Protocol Review Committee (YRC-PRC) Approval Date:

*Dept. of Veterans Affairs, West Haven VA HSS Approval Date:

*Radioactive Drug Research Committee (RDRC) Approval Date:

YNHH-Radiation Safety Committee (YNHH-RSC) Approval Date:

Yale University RSC (YU-RSC) Approval Date:

Magnetic Resonance Research Center PRC (MRRC-PRC) Approval Date:

*Nursing Research Committee Approval Date:

YSM/YNHH Cancer Data Repository (CaDR) Approval Date:

Dept. of Lab Medicine request for services or specimens form

Imaging on YNHH Diagnostic Radiology equipment request form (YDRCTO request) found at [http://radiology.yale.edu/research/ClinTrials.aspx](%20http:/radiology.yale.edu/research/ClinTrials.aspx))

****Approval from these committees is required before final HIC approval is granted. See instructions for documents required for initial submission and approval of the protocol. Allow sufficient time for these requests. Check with the oversight body for their time requirements.***

1. **Probable Duration of Project:** State the expected duration of the project, including all follow-up and data analysis activities. 12/1/2016-11/30/2021
2. **Research Type/Phase: (Check all that apply)**

a. **Study Type**

Single Center Study

Multi-Center Study

Does the Yale PI serve as the PI of the multi-site study? Yes  No

Coordinating Center/Data Management

Other:

b. **Study Phase**  **N/A**

Pilot  Phase I  Phase II  Phase III  Phase IV

Other (*Specify)*

1. **Area of Research: (Check all that apply)** Note that these are overlapping definitions and more than one category may apply to your research protocol. Definitions for the following can be found in the instructions section 4c:

Clinical Research: Patient-Oriented  Clinical Research: Outcomes and

Clinical Research: Epidemiologic and Behavioral Health Services

Translational Research #1 (“Bench-to-Bedside”)  Interdisciplinary Research

Translational Research #2 (“Bedside-to-Community”)  Community-Based Research

5. Is this study a clinical trial? Yes  No

*NOTE the current ICMJE (International Committee of Medical Journal Editors) definition of a clinical trial: “any research study that prospectively assigns human participants or groups of humans to one or more health-related interventions to evaluate the effects on health outcomes.” Health-related interventions include any intervention used to modify a biomedical or health-related outcome (for example, drugs, surgical procedures, devices, behavioral treatments, dietary interventions, and process-of-care changes). Health outcomes include any biomedical or health-related measures obtained in patients or participants, including pharmacokinetic measures and adverse events”*

If yes, where is it registered?

Clinical Trials.gov registry  will be registered with ClinicalTrials.gov

Other (*Specify*)

*Registration of clinical trials* ***at their initiation*** *is required by the FDA, NIH and by the ICMJE.*

*If this study is registered on clinicaltrials.gov, there is new language in the consent form and compound authorization that should be used.*

For more information on registering clinical trials, including whether your trial must be registered, see the YCCI webpage, <http://ycci.yale.edu/researchers/ors/registerstudy.aspx> or  contact YCCI at 203.785.3482)

1. Does the Clinical Trials Agreement (CTA) require compliance with ICH GCP (E6)?

Yes  No

7. Will this study have a billable service? *A billable service is defined as any service rendered to a study subject that, if he/she was not on a study, would normally generate a bill from either Yale-New Haven Hospital or Yale Medical Group to the patient or the patient’s insurer. The service may or may not be performed by the research staff on your study, but may be provided by professionals within either Yale-New Haven Hospital or Yale Medical Group (examples include x-rays, MRIs, CT scans, specimens sent to central labs, or specimens sent to pathology). Notes: 1. There is no distinction made whether the service is paid for by the subject or their insurance (Standard of Care) or by the study’s funding mechanism (Research Sponsored). 2. This generally includes new services or orders placed in EPIC for research subjects*.

Yes  No All data will be collected in Ukraine

If answered, “yes”, this study will need to be set up in OnCore, Yale’s clinical research management system, for Epic to appropriately route research related charges. Please contact [oncore.support@yale.edu](mailto:oncore.support@yale.edu)

8.. Are there any procedures involved in this protocol that will be performed at YNHH or one of its affiliated entities? Yes ___ No __X_ *If Yes, please answer questions a through c and note instructions below. If No, proceed to Section III.*

a. Does your YNHH privilege delineation currently include the **specific procedure** that you will perform? N/A

b. Will you be using any new equipment or equipment that you have not used in the past for this procedure?

c. Will a novel approach using existing equipment be applied?

If you answered “no” to question 8a, or "yes" to question 8b or c, please contact the YNHH Department of Physician Services (688-2615) for prior approval before commencing with your research protocol.

*Please note that if this protocol includes Yale-New Haven Hospital patients, including patients at the HRU, the Principal Investigator and any co-investigators who are physicians or  mid-level practitioners (includes PAs, APRNs, psychologists and speech pathologists) who may have direct patient contact with patients on YNHH premises must have medical staff appointment and appropriate clinical privileges at YNHH. If you are uncertain whether the study personnel meet the criteria, please telephone the Physician Services Department at 203-688-2615.* ***By signing this protocol as a PI, you attest that you and any co-investigator who may have patient contact has a medical staff appointment and appropriate clinical privileges at YNHH****.*

## Section III: Funding, research team and training

1. **Funding Source:** Indicate all of the funding source(s) for this study. Check all boxes that apply.

Provide information regarding the external funding source. This information should include identification of the agency/sponsor, the funding mechanism (grant or contract), and whether the award is pending or has been awarded. Provide the M/C# and Agency name (if grant-funded). If the funding source associated with a protocol is “pending” at the time of the protocol submission to the HIC (as is the case for most NIH submissions), the PI should note “Pending” in the appropriate section of the protocol application, provide the M/C# and Agency name (if grant-funded) and further note that University (departmental) funds support the research (until such time that an award is made).

| **PI** | **Title of Grant** | **Name of Funding Source** | **Funding** | **Funding Mechanism** |
| --- | --- | --- | --- | --- |
| Frederick Altice, MD | Integrating Addiction Treatment and HIV Services into Primary Care Clinics in Ukraine | NIDA | Federal  State  Non Profit  Industry  Other For Profit  Other | Grant-M# pending  Contract#  Contract Pending  Investigator/Department Initiated  Sponsor Initiated  Other, Specify: |
|  |  |  | Federal  State  Non Profit  Industry  Other For Profit  Other | Grant-M#  Contract#  Contract Pending  Investigator/Department Initiated  Sponsor Initiated  Other, Specify: |
|  |  |  | Federal  State  Non Profit  Industry  Other For Profit  Other | Grant-M#  Contract#  Contract Pending  Investigator/Department Initiated  Sponsor Initiated  Other, Specify: |

IRB Review fees are charged for projects funded by Industry or Other For-Profit Sponsors. Provide the Name and Address of the Sponsor Representative to whom the invoice should be sent. ***Note: the PI’s home department will be billed if this information is not provided.***

**Send IRB Review Fee Invoice To:**

Name:

Company:

Address:

1. **Research Team:**  List all members of the research team. Indicate under the affiliation column whether the investigators or study personnel are part of the Yale faculty or staff, or part of the faculty or staff from a collaborating institution, or are not formally affiliated with any institution. **ALL members of the research team MUST complete Human Subject Protection Training (HSPT) and Health Insurance Portability and Accountability Act (HIPAA) Training before they may be listed on the protocol. See NOTE below.**

|  | **Name** | **Affiliation: Yale/Other Institution (Identify)** | **NetID** |
| --- | --- | --- | --- |
| **Principal Investigator** | Frederick Altice | Yale | FA7 |
| **Role: Co-I** | David Paltiel | Yale |  |
| **Role: Co-I** | Denise Esserman | Yale |  |
| **Role: Program Manager** | Ruthanne Marcus | Yale | RM254 |
| **Role: Project Coordinator** | Trena Mukherjee | Yale | TIM4 |
| **Role: ARS** | Julia Rozanova | Yale |  |
| **Role: Data Manager** | Maua Herme | Yale | MH535 |
| **Role: Biostatistician** | Fangyong Li | Yale |  |
| **Role: Site PI** | Sergii Dvoriak | Non-Yale; UIPHP |  |

**NOTE: The HIC will remove from the protocol any personnel who have not completed required training. A personnel protocol amendment will need to be submitted when training is completed.**

## Section IV:

## Principal Investigator/Faculty Advisor/ Department Chair Agreement

As the **principal investigator** of this research project, I certify that:

- The information provided in this application is complete and accurate.
- I assume full responsibility for the protection of human subjects and the proper conduct of the

research.

- Subject safety will be of paramount concern, and every effort will be made to protect subjects’

rights and welfare.

- The research will be performed according to ethical principles and in compliance with all federal,

state and local laws, as well as institutional regulations and policies regarding the protection of

human subjects.

- All members of the research team will be kept apprised of research goals.
- I will obtain approval for this research study and any subsequent revisions prior to my initiating the

study or any change and I will obtain continuing approval of this study prior to the expiration date

of any approval period.

- I will report to the HIC any serious injuries and/or other unanticipated problems involving risk to

participants.

- I am in compliance with the requirements set by the [University](http://www.yale.edu/provost/handbook/handbook_x__university_policies_concerni.html#T2) and qualify to serve as the

principal investigator of this project or have acquired the appropriate approval from the

Dean’s Office or Office of the Provost, or the Human Subject Protection Administrator at

Yale-New Haven Hospital, or have a faculty advisor.

- I will identify a qualified successor should I cease my role as principal investigator and facilitate a

smooth transfer of investigator responsibilities.

_____

PI Name (PRINT) and Signature Date

As the **faculty advisor** of this research project, I certify that:

- The information provided in this application is complete and accurate.
- This project has scientific value and merit and that the student or trainee investigator has the necessary resources to complete the project and achieve the aims.
- I will train the student investigator in matters of appropriate research compliance, protection of human subjects and proper conduct of research.
- The research will be performed according to ethical principles and in compliance with all federal, state and local laws, as well as institutional regulations and policies regarding the protection of human subjects.
- The student investigator will obtain approval for this research study and any subsequent revisions prior to initiating the study or revision and will obtain continuing approval prior to the expiration of any approval period.
- The student investigator will report to the HIC any serious injuries and/or other unanticipated problems involving risk to participants.
- I am in compliance with the requirements set forth by the [University](http://www.yale.edu/provost/handbook/handbook_x__university_policies_concerni.html#T2) and qualify to serve as the faculty advisor of this project.
- I assume all of the roles and responsibilities of a Principal Investigator even though the student may be called a PI.

___________ ______ _____

Advisor Name (PRINT) and Signature Date

Signature of PI Date

**Department Chair’s Assurance Statement**

Do you know of any real or apparent institutional conflict of interest (e.g., Yale ownership of a

sponsoring company, patents, licensure) associated with this research project?

Yes (provide a description of that interest in a separate letter addressed to the HIC.)

No

As Chair, do you have any real or apparent protocol-specific conflict of interest between yourself and

the sponsor of the research project, or its competitor or any interest in any intervention and/or method

tested in the project that might compromise this research project?

Yes (provide a description of that interest in a separate letter addressed to the HIC)

No

I assure the HIC that the principal investigator and all members of the research team are qualified by

education, training, licensure and/or experience to assume participation in the conduct of this research

trial. I also assure that the principal investigator has departmental support and sufficient resources to

conduct this trial appropriately.

____________________________

Chair Name (PRINT) and Signature Date

_________________________________

Department

**YNHH Human Subjects Protection Administrator Assurance Statement**

*Required when the study is conducted solely at YNHH by YNHH health care providers.*

As Human Subject Protection Administrator (HSPA) for YNHH, I certify that:

- I have read a copy of the protocol and approve it being conducted at YNHH.
- I agree to notify the IRB if I am aware of any real or apparent institutional conflict of interest.
- The principal investigator of this study is qualified to serve as P.I. and has the support of the hospital for this research project.

______________________________________

YNHH HSPA Name (PRINT) and Signature Date

## Section V: Research Plan

1. **Statement of Purpose:** State the scientific aim(s) of the study, or the hypotheses to be tested.

**1)** To compare both primary (composite QHI score) and secondary (individual HIV/MMT/TB/PC QHI scores, quality of life, and stigma) outcomes in 1,350 HIV+ PWIDs receiving MMT from 15 regions (clusters) and 45 clinical settings using a stratified, phase-in, cluster-controlled design over 24 months. After stratifying HIV+ PWIDs based on their current receipt of MMT, they will be randomized to receive MMT in specialty addiction clinics (N=450) or in an *ECHO-IC/QI-enhanced* primary care clinic with (N=450) or without (N=450) P4P incentives;

**2)** Using a multi-level implementation science framework, to examine the contribution of client, clinician and organizational factors that contribute to the comprehensive composite (primary outcome) and individual (secondary) QHI scores; and

**3)** To conduct a cost-effectiveness analysis (CEA) of integrating HIV/MMT into PC sites, with or without P4P, compared to a control group of specialized MMT sites.

1. **Background:** Describe the background information that led to the plan for this project. Provide references to support the expectation of obtaining useful scientific data.

Ukraine’s volatile HIV epidemic, the worst in Europe, is fueled primarily by opioid-dependent people who inject drugs (PWIDs).[^1-3^](#_ENREF_1) High HIV prevalence (21.3%-41.8%) in PWIDs[^4^](#_ENREF_4) account for >70% of cumulative and >56% of new HIV infections.[^5^](#_ENREF_5) The Institute of Medicine (IOM)[^6^](#_ENREF_6) and WHO/UNAIDS Technical Guide *for countries to set targets for universal access to HIV prevention, treatment and care for PWID* prioritizes methadone maintenance therapy (MMT) scale-up as a core HIV prevention and treatment strategy,[^7^](#_ENREF_7) including within primary care (PC). Modeling for Ukraine shows that MMT scale-up is the *most cost-effective* HIV prevention strategy, but when combined with antiretroviral therapy (ART) as ‘treatment as prevention’, is more effective but at 2.5-fold increased cost.[^8^](#_ENREF_8) MMT (2.7%)[^9^](#_ENREF_9)^,^[^10^](#_ENREF_10) and ART (4%) coverage[^10^](#_ENREF_10) for the ~310,000 PWIDs remains low since we introduced buprenorphine in 2004,[^11^](#_ENREF_11) MMT in 2008,[^12^](#_ENREF_12)^,^[^17^](#_ENREF_17) and integrated MMT into specialty clinics in 2010.[^13^](#_ENREF_13) MMT scale-up is hampered by client, clinician and structural barriers;[^14-19^](#_ENREF_14) however, many can be overcome by integrating MMT into primary care (PC).[^14^](#_ENREF_14) Expected international funding reductions (2017) prompted new healthcare reform and financing efforts by reducing emphasis on specialty care and strengthening PC for medically complex people living with HIV (PLH) who are PWID. In our PEPFAR pilot study based on the Collaborative Care Model (CCM),[^20-29^](#_ENREF_20) we confirmed that integrating MMT into PC was acceptable, feasible, convenient, reduced stigma and effectively managed medical comorbidities (HIV, TB, HCV, depression, etc.) that was not otherwise handled in MMT specialty clinics.

Despite international recommendations to integrate MMT, HIV and PC services,[^30-33^](#_ENREF_30) there is little empirical guidance for administrators, clinicians, policy makers and funders. Real-world demonstration projects with PWIDs and empirical findings from RCTs would provide new guidance for MMT integration for PLH in PC settings. Successful integration requires understanding ‘how’ integration improves non-addiction treatment outcomes (e.g., HIV, TB, etc) using objective composite quality health indicators (QHI), which are increasingly used to measure healthcare improvements comprehensively.[^13^](#_ENREF_13)^,^[^34-36^](#_ENREF_34) QHIs as an outcome is especially salient since addiction patients treated in specialty settings, on average, die 25 years before their peers, due to suboptimal treatment of co-morbid conditions.[^37^](#_ENREF_37)^,^[^38^](#_ENREF_38) QHIs are therefore innovative measures that apply recommended clinical care standards to measure healthcare integration factors across a spectrum of addiction and non-addiction comorbidities, and are increasingly used in quality improvement (QI) processes, including pay-for-performance (P4P) healthcare financing reforms that are increasingly being used to strengthen primary care.[^39^](#_ENREF_39) P4P strategies, with mixed findings from variably designed studies,[^40-42^](#_ENREF_40) are recommended by the IOM,[^43^](#_ENREF_43) and provide pre-specified incentives to clinicians to manage patient and can strengthen healthcare delivery.

Project **ECHO** (**E**xtension for **C**ommunity **H**ealthcare **O**utcomes), an evidence-based practice (EBP) that improves treatment outcomes for specialty conditions within non-specialty settings, uses a collaborative learning environment to continuously train, coach, and reinforce *specialty* care practices (e.g., managing comorbidities) for non-specialist physicians using tele-education technology. It provides interactive, continuous learning,[^44^](#_ENREF_44)^,^[^45^](#_ENREF_45) skill-building and care management (including QI methods) that empowers non-specialist clinicians to provide specialty treatment that is traditionally not within their scope of practice. To address the multiple syndemic medical comorbidities of HIV+ PWIDs in Ukraine, we will create and test innovations to integrate specialty services (e.g., managing MMT, HIV, TB, HCV) into PC using the Project ECHO – Integrated Care (**ECHO-IC**)/QI-enhanced PC model where ECHO-IC clinical skills will be *enhanced* with QI methods to coach and assist clinicians to improve fidelity to clinical care standards and assess their performance to achieve recommended clinical care standards either with or without evidence-based P4P financial incentives.

1. **Research Plan:** Summarize the study design and research procedures using non-technical language that can be readily understood by someone outside the discipline. **Be sure to distinguish between standard of care vs. research procedures when applicable, and include any flowcharts of visits specifying their individual times and lengths**. Describe the setting in which the research will take place.

**FROM GRANT APPLICATION:**

**III.C.1. Aim 1:** To compare both primary (composite QHI score) and secondary (individual HIV/MMT/TB/PC QHI scores, quality of life, and stigma) outcomes in 1,350 HIV+ PWIDs receiving MMT from 15 regions (clusters) and 45 clinical settings using a stratified, phase-in, cluster-controlled design over 24 months. After stratifying PWIDs based on current receipt of MMT, they will be randomized to receive MMT in specialty addiction clinics (N=450) or in an *ECHO-IC/QI-enhanced* primary care clinic with (N=450) or without (N=450) P4P incentives.

C.1.a. Hypotheses: 1) PWIDs receiving MMT in PC will have significantly higher QHIs than PWIDs in specialty MMT sites; 2) composite and individual QHI scores will increase for levels of service (P4P>No P4P>addiction sites); 3) PWIDs receiving MMT in PC will have significantly lower stigma than PWIDs in specialty MMT sites.


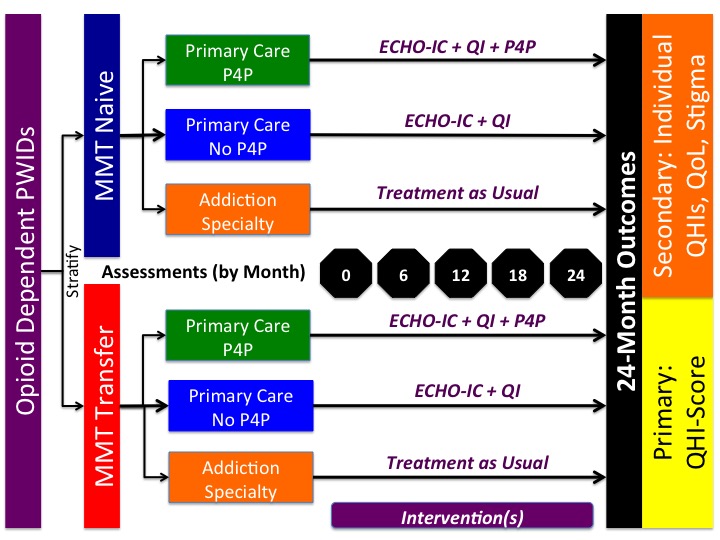
C.1.b. Study Design: See adjacent figure where we have selected the 15 highest burden oblasts (regions) for both PWIDs and HIV. Based on our preliminary studies suggesting site-specific differences toward HIV/MMT integration and because each healthcare region is modestly autonomous and may prioritize healthcare delivery differentially, we have chosen to “cluster” each of the three interventions within the same geographic region. Nationally, 94% of PWIDs use opioids. All stratification and randomization will be clustered within each oblast (administratively different). From within each oblast, the largest MMT site plus two PC sites will be selected; PC sites will be decided based on our preliminary data from ExMAT mapping where the most HIV/PWID cases reside. We will randomly select 5 oblasts for each phase-in, based on number of PWIDs and HIV prevalence to ensure that each “phase” is similarly matched. All recruited HIV+ PWIDs will be stratified 1:1 as MMT transfer or MMT naive. Rationale for transferring newly initiated MMT patients to PC sites is that it provides an opportunity for MMT sites to transfer more stabilized patients and initiate new ones. Rationale for new MMT patients is to observe PC clinician competency with new MMT initiation. All recruitment will occur at MMT specialty sites for treatment-seeking patients. For both the MMT transfer and new participants, they will be randomized to receive MMT at either the MMT specialty site (orange), PC without P4P (blue), or PC with P4P (green), with equal allocation. Historically PLH have been prioritized for MMT, which will allow us to access enough patients to complete enrollment as planned. The phase-in will include 5 regions that will initiate enrollment of patients over a 6-month period, which allows sites to organize, be trained and allow them to address logistical constraints of implementation. Participants, providers and sites will be assessed at baseline and every 6 months over 24 months.

C.1.c. Eligibility criteria: ≥18 years, HIV+, ICD-10 criteria for opioid dependence & interested in or on MMT <90 days, reside within the legal clinical catchment area to receive PC and not be under police investigation or planning to move.

C.1.d. Allocation: Recruitment of recently initiated PLH on MMT and new MMT patients will be 1:1. *MMT naïve* patients seek MMT at the addiction specialty site, where they will undergo randomization 1:1:1 to the three conditions. We will employ stratified (region and MMT status), permuted block randomization with variable block sizes.

C.1.e. Interventions: All participants will be linked to a PCP. The standard of care control (addiction specialty) will include enrollment in MMT as is currently done nationally, though each oblast may have unique criteria and regulations. National regulations require MMT administration as DOT within an approved site. PC sites (+/- P4P) will have two dedicated MDs and one RN who oversee MMT. PC sites will have access to & training for point-of-care testing for HIV, HCV and TB (Gene Xpert), to promote onsite service integration. Clinical staff will receive two 3-day training sessions. One will consist of core competencies on integrated care (e.g., MMT, HIV, TB, hepatitis) using Regional Learning Hub resources and how to effectively participate in ECHO-IC. The second 3-day training session will cover QI skills and procedures. QI training will introduce QI as a practice strategy to improve delivery of evidence-based care for PWIDs (and more generally). This 3 day training will take place prior to study initiation, and will be facilitated by Yale and UIPHP staff. PC staff will also receive guidance on weekly ECHO activities. Three weekly ECHO-IC “clinical” sessions will be followed that include both didactic and case-based learning followed by one “QI” session that uses a similar format, including case examples. P4P sites will know upfront that there is a ‘pool’ of money that can be earned annually as a bonus, based on whether their patients achieve clearly stated care standards (i.e. QHI score). Clinicians that achieve these care standards will divide the bonus accordingly.

C.1.f. Retention: We will use several steps used in the pilot study and with other studies of PWIDs where retention was 94% in the pilot and in all cases exceeds 83% in other studies, including: 1) providing compensation time for travel time and assessment costs; 2) detailed locator information; 3) maintaining regular contact through MMT DOT activities.

C.1.g. Assessments: All 1,350 subjects will undergo assessments at baseline and every 6 months for 24 months. Protocol activities and assessments will be modestly adapted from the Pilot Protocol in the appendix. Before allocation, all subjects will sign a release of information (medical and criminal justice) form to allow research staff to access their medical records. Research staff will access records both in their allocated treatment center and other clinics or healthcare facilities where the patient indicates that they have received care. At the start of the assessment, patients will be asked to provide the types of healthcare services received within the past six months, and the location of the clinic where the service was received. Research staff will then call the listed clinics to verify the type and date of each service. Surveys in the remainder of the assessment will include demographics, geospatial assessment, addiction severity (DAST-10),[^159^](#_ENREF_159) alcohol use disorders (AUDIT-C), MMT readiness (SOCRATES),[^160^](#_ENREF_160)^,^[^161^](#_ENREF_161) MMT treatment experience (satisfaction, barriers & facilitators, adherence), HIV risk behaviors (modified RBA),[^162^](#_ENREF_162) medical comorbidity and health assessment (current and past health service utilization), depressive symptoms (CES-D),[^163^](#_ENREF_163) status and treatments for HIV, HBV, HCV, TB and STIs, access to and use of healthcare services (including paid expenses), QoL (SF-12),[^164^](#_ENREF_164) trust in physician scale,[^165^](#_ENREF_165)^,^[^166^](#_ENREF_166) stigma (HIV[^167^](#_ENREF_167) and Drug Use[^168^](#_ENREF_168)),[^167^](#_ENREF_167) incarceration, and police detention and harassment experiences. Standardized instruments with high psychometric properties were selected from NIDA’s Data Harmonization process (Altice, lead for addiction, HIV risk behaviors and adherence workgroups), allowing future comparisons to other domestic and international settings. MMT retention will be measured from DOT records. Medical chart review will assess QHIs and supplemented by patient self-report (validated previously with Pearson r=0.84).[^35^](#_ENREF_35)^,^[^169^](#_ENREF_169) The MoH retains all healthcare utilization visits and testing visits at the MoH’s Chief Medical Officer for each region and the Ukrainian CDC maintains all MMT data (which we developed as part of ExMAT).

C.1.h. Research Quality Assessment: We will employ several strategies used in ExMAT and other trials to ensure fidelity and monitor “intervention drift” among providers delivering HIV/MMT care including: 1) *Training of providers*: We will provide a 3-day clinical competency training on MMT, HIV, TB, HBV, HCV followed by a 3-day QI training before implementing ECHO-IC; 2) *Clinic Site visits*: Our Project Coordinator will schedule site visits every 6 months to meet with clinicians and administrators at each site to discuss challenges and their experiences in delivering MMT and assess quality and fidelity of implementing the intervention. We will use methods used in our pilot that evolved from BHIVES where we assessed organizational factors based on what’s working, not working and goals for improvement;[^67^](#_ENREF_67) 3) *Technical Assistance*: Clinic providers will be able to access technical assistance weekly from ECHO-IC and from our experts by phone and email on an ongoing basis, which will be monitored and assessed; 4) *Quality Improvement*: All ECHO and QI sessions and site visits will be digitally recorded and will be reviewed for supervision by experienced QI coaches and HQI mentors; 5) *Regular review of process measure data*: Rapid collection of process measures via our electronic data management system will allow us to monitor fidelity of implementation of intervention and allow quick feedback.

C.1.i. Procedures to Maximize Internal and External Validity: Additional measures will monitor treatment contamination, exposure to outside medical services (HIV, TB, harm reduction, etc), and social desirability threats that may compromise internal validity. *Treatment Contamination*: To minimize the threat of contamination, providers will be trained about the experimental nature of the intervention and importance of not sharing findings. If any patient should move to a new polyclinic catchment area, we will gather this as during interviews or chart review. Preliminary data suggest patients rarely move to new neighborhoods but monitor through quarterly retention contacts. During quarterly site visits, the Project Director will meet with providers to identify and address potential contamination threats between MMT patients (i.e. moving site of care). All participants will complete the Diffusion Questionnaire at each follow-up that contains six items asking if they discussed any information that they learned with other participants. *Exposure to Outside Addiction, HIV or TB HIV services*: A subsection of semi-annual survey will measure exposure to outside services. Type and date of referral contacts for each participant will also be collected from providers. *Social Desirability*: Participants will complete a standard measure of social desirability bias[^170^](#_ENREF_170) to examine whether intervention effects can be explained by such bias.

C.1.j. Data sources and management: All patient and staff surveys will be collected using study ID via Qualtrics and QHIs will be collected using chart review and entered onto clinical research forms. All data are uploaded directly into REDCap with clerical checking and patient safeguards described in detail in the human subjects section.

**III.C.2. Aim 2:** Using a multi-level implementation science framework, we will examine the contribution of client-level, clinician-level and organizational-level factors that contribute to the comprehensive QHI score.


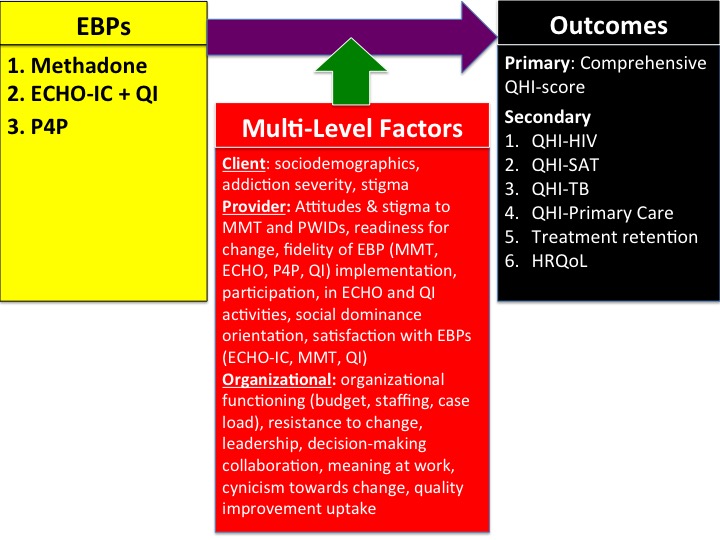
C.2.a. Conceptual Framework: The adjacent figure provides the conceptual framework that incorporates diffusion of innovation theory[^147^](#_ENREF_147) and implementation research.[^148-151^](#_ENREF_148) This innovative framework serves as a comprehensive heuristic model that may identify different multi-level characteristics that may influence MMT integration into PC and on QHIs, including: 1) Client-level factors that may help identify how different subgroups of clients respond to the intervention arms; 2) Provider-level factors that emphasize factors related to the characteristics of the staff, their attitudes toward MMT and PWIDs, and fidelity of implementation of MMT, ECHO-IC with QI participation, & P4P; 3) Organizational-level factors that emphasize organizational characteristics of the clinic site (e.g., budget, caseload, size) and more broadly clinical staff attitudes toward change and uptake of QI activities. Community-level factors that include local regulations, stigma/discrimination, policing, drug-related policies, institutional response towards accepting an integrated model will be assessed based on and controlled for by region (cluster). To examine the influence of multi-level factors on implementation of intervention, we will employ a mixed methods approach collecting quantitative (see aim 1) and qualitative data to evaluate the influence of client-level, provider-level, organizational, community-level and structural factors that impede or facilitate the implementation of MMT and effectiveness of study arm interventions. Quantitative data on *changes* in the multi-level factors (client, provider, organization, and community) over time and how they influence the effectiveness of MMT integration on QHIs (e.g., recruitment, retention, fidelity of implementation) will be compared and contrasted to key themes that emerge from the qualitative data. By merging these data we can more accurately interpret study findings if one of the intervention arms fails to meet aim 1 hypotheses and together these data will inform dissemination strategies to scale-up MMT more broadly across Ukraine, Eastern Europe and Central Asia that have similar healthcare structures.

C.2.b. Qualitative Assessments: The qualitative data will help us identify the real-world contexts and different organizational, community and structural factors that promote or impede the successful MMT integration into PC, which would otherwise be unobserved. From each of 45 sites, we will randomly select and use semi-structured in-depth interviews with 3 clients (N=135 clients), 2 staff members (N=90 staff), the Chief Narcologist for each oblast (N=15) and one administrator from each PC site (n=30). A structured interview guide will be used (see Pilot Protocol documents in Appendix) with clients to elicit their feedback on the relevance of MMT delivery and how it influenced their engagement and retention in MMT and PC care, adherence to health recommendations, barriers to PC, interactions with clinicians and police, and peer norms about MMT. Interviews with clinicians will elicit their feedback on training and technical assistance using ECHO-IC, their perceptions about the relevance of MMT, ECHO and P4P, and perceptions of organizational climate and organizational factors that may facilitate or impede their ability to deliver MMT. Interviews with clinic site administrators will assess their perceptions of organizational factors that facilitate or impede MMT integration, ECHO, QI and P4P as well as their observations about how the integration is proceeding. All interviews will be audio-recorded, transcribed, translated into English and back-translated to ensure accurate interpretation.[^174^](#_ENREF_174) Grounded theory will be used to explore and group themes using Atlas 5.2 software system. A preliminary set of analytic coding categories (closed codes) will be assembled based on concepts, themes and patterns. This set of codes will be updated through a process of contrast and comparison. Key themes related to how different factors may influence the delivery of the intervention will be compared to the quantitative study results for Aim 2 and fidelity of implementation data. Themes that emerge will be put into an organizational matrix to observe changes that occur during integration and participation in ECHO-IC and QI activities.

C.2.c. Quantitative Assessment of Organizational Factors: Staff and administrators will complete a survey at baseline and every 6 months. Provider-level factors will include provider characteristics (e.g., socio-demographics, education and work experience) and attitudes toward MMT, P4P, & QI, attitudes toward an integrated care model of care and their level of self-efficacy implementing MMT. Attitudes and stigma towards drug users will be operationalized as stereotypical beliefs, attribution beliefs, expectations with regard to rehabilitation chances and social distance,[^175^](#_ENREF_175) and previously used to compare attitudes between PC and addiction specialists.[^176^](#_ENREF_176)^,^[^177^](#_ENREF_177) For Organizational Factors, clinic administrators will be asked to complete brief surveys on organizational characteristics (number, type, and turnover of staff, types of services provided, hours spent on clinical and administrative tasks, budget). We will also use the validated HQI facility level organizational assessment (see appendix) that provides clinic and national level leaders with information on site-level achievements. Routine organizational assessments to gauge the progress of implementation and identify areas for future growth are conducted initially by improvement coaches with transfer of implementation to the country team. These results will be reviewed by the MoH, regional health directors with support from QI coaches. This tool supports continued learning and comprehension and promotes higher level expertise on the science of QI implementation more broadly as a strategy to improve population health. Key domains include: leadership support, QI committees, quality planning, workforce engagement and QI involvement, use of data for improvement, QI implementation and competency in QI methods, formal patient involvement mechanisms, evaluation, and outcomes. Results are used to develop a workplan for each element with specific action steps and timelines guiding the process: to set prioritized direction and ensure that resources are allocated for the local QI program. Results of the tool will be communicated to internal key stakeholders, leadership, and staff, whose engagement buy-in is essential for translating results into improvement practice and analyzed as repeated measures. To understand organizational change, we will also have clinic staff and administrators complete several brief validated scales twice annually using Qualtrics where we have had high response (>85%): Organizational Capacity Inventory (e.g., perceived organizational climate, norms, trust and communication) and Organizational Readiness to Change Scale[^178^](#_ENREF_178) and Organizational Functioning Scale[^178^](#_ENREF_178). These brief organizational measures include standardized scales for Social Dominance Orientation,[^179^](#_ENREF_179) which assesses the differences in how staff perceive themselves as “superior” to their patients and often observed in addiction treatment (α>0.90); Resistance Towards Change,[^180^](#_ENREF_180)^,^[^181^](#_ENREF_181) which assesses how staff respond to innovation within an organization (α>0.90). Discrimination toward PWIDs by staff will be measured using the Punishment Scale,[^182^](#_ENREF_182) which assesses the extent to which staff members believe that individuals should be punished for their drug use and related activities (α>0.85; including subscales) and WHO’s Intolerance to Addiction Scale,[^183^](#_ENREF_183) that assesses staff attitudes toward addiction; as well as additional attitudes that we have created that include information, motivation and necessary skills needed for integration of addiction into PC settings and towards provision of MMT. A review of policies and procedures (and changes) by the HQI staff will be used to assess Community-level Factors, including types of services available, geographic proximity of treatment and prevention services, and perceived barriers from patients to accessing services (e.g., hours of service, transportation) from aim 1. Structural factors will include indicators of policing (i.e. frequency of arrests); incarceration, stigma and discrimination experienced by participants.

**III.C.3. Aim 3**: To conduct a cost-effectiveness analysis (CEA) of integrating HIV/MMT into PC sites, with or without P4P, compared to a control group of specialized MMT sites.

C.3.a. Overview: We will employ a dynamic, model-based approach to estimate the cost-effectiveness of integrating HIV/MMT into PC. By capturing non-linearity in the transmission of infections and behaviors resulting from dependence of outcomes among individuals, we will be able to understand and evaluate population-level benefits and associated costs in the context of HIV and other comorbid conditions.[^189^](#_ENREF_189) The analysis will be conducted in accordance with the recommendations of the U.S. Panel on Cost-Effectiveness in Health and Medicine. We will parameterize the model using program-level cost and outcome data, supplemented by data obtained from the pilot study and country-level data obtained from published and publicly available sources. We will report comparative value in terms of incremental cost-effectiveness ratios (ICER).[^190^](#_ENREF_190) The principal health outcome of interest will be the QHIs, however, we will also analyze data using quality adjusted life-years (QALYs) as an outcome to enable comparison with other CEA studies. We will also examine the impact of the 3 interventions on the number of averted HIV infections (in the population-level dynamic model). We will adopt the modified societal perspective, taking into account both direct costs and benefits to individual patients and population-level transmission and economic effects. We will report all economic outcomes in inflation-adjusted, 2016 US dollars and discount all outcomes at an annual rate of 3% per year.[^190^](#_ENREF_190) We will apply the cost-effectiveness threshold standards of the WHO Commission on Macroeconomics and Health, which suggests that interventions be judged in terms of a given country’s ability to pay, as measured by their national per capita gross domestic product (GDP). We will define a strategy as “very cost-effective” if its ICER is less than the Ukrainian GDP (~US$7,000) and “cost-effective” if its ICER is less than 3 times the GDP (~US$21,000). In managing the uncertainty and exploring the robustness of our analysis, we will adhere to the recommendations of the ISPOR-SMDM Modeling Good Research Practices Task Force and, in particular, its report on Model Parameter Estimation and Uncertainty, employing both deterministic and probabilistic methods of sensitivity analysis, as appropriate and feasible.

Individual survival (and quality-adjusted survival) gains are associated with increased MMT coverage based on reduced injection and mortality, blood-borne infections, criminality, and increased QoL, access to treatment for HIV, TB and other preventable or treatable conditions, and employment. Comparisons will be made based on QHIs achieved for the status quo compared to integrated care +/- P4P. For population-level outcomes, in addition to direct patient-level QALY gains calculated, there is expected to be indirect population-level QALY gains due to non-linearity of transmission dynamics from reductions in drug injection, improved access to ART, TB prevention and treatment, HCV care, and PC management, but balanced by increased survival that might result in increased transmission from sex and injection risks.

1. **Genetic Testing N/A**
2. **Subject Population:** Provide a detailed description of the types of human subjects who will be recruited into this study.

In Aim 1 we will enroll HIV+ PWID seeking MMT. As part of Aim 2, in addition to the HIV+ study participants seeking MMT, we will interview and survey clients, clinicians and health center administrators from participating primary care centers. In our program, ECHO-IC (Integrated Care), we will supplement the ECHO-based clinical skills with Quality Improvement (QI) techniques to assist with improving fidelity to clinical care standards and provide PC physicians with QI skills to assess to performance and further motivate improved recommended clinical practices that link evidence-based financial incentives using pay-for-performance (P4P) strategies. Aim 3 involves a cost-effectiveness analysis (CEA) of data collected in the first two Aims.

1. [**Subject classification:**](file://C:\Users\mml37\AppData\Local\Microsoft\Windows\Temporary%20Internet%20Files\Content.Outlook\AppData\Local\Microsoft\Windows\Documents%20and%20Settings\jhl3\Local%20Settings\Temporary%20Internet%20Files\Content.Outlook\Local%20Settings\Temporary%20Internet%20Files\cmm82\Local%20Settings\Temporary%20Internet%20Files\Regulatory%20Review%20Comments%20ML.JM\100%20FR%201a%20HIC%20Protocol_Application_Instructions%2006-21-10.doc#Subjects) Check off all classifications of subjects that will be specifically recruited for enrollment in the research project. Will subjects who may require additional safeguards or other considerations be enrolled in the study? If so, identify the population of subjects requiring special safeguards and provide a justification for their involvement.

Children  Healthy Fetal material, placenta, or dead fetus

Non-English Speaking  Prisoners  Economically disadvantaged persons

Decisionally Impaired  Employees  Pregnant women and/or fetuses

Yale Students Females of childbearing potential

People who inject drugs.

NOTE: Is this research proposal designed to enroll children who are wards of the state as potential subjects?  Yes  No (If yes, see Instructions section VII #4 for further requirements)

1. [**Inclusion/Exclusion Criteria**](file://C:\Users\mml37\AppData\Local\Microsoft\Windows\Temporary%20Internet%20Files\Content.Outlook\AppData\Local\Microsoft\Windows\Documents%20and%20Settings\jhl3\Local%20Settings\Temporary%20Internet%20Files\Content.Outlook\Local%20Settings\Temporary%20Internet%20Files\cmm82\Local%20Settings\Temporary%20Internet%20Files\Regulatory%20Review%20Comments%20ML.JM\100%20FR%201a%20HIC%20Protocol_Application_Instructions%2006-21-10.doc#eligibility)**:** What are the criteria used to determine subject inclusion or exclusion?

Inclusion criteria: For the ECHO-IC, we will enroll PWID interested in MMT, 18 years of age or older, meeting DSM-V criteria for opioid dependence. Eligible participants will either be treatment naïve and seeking MMT or will be on MMT <90 days. For Structured Staff surveys and the Qualitative Assessments, we will include recruitment of clients, staff, and administrators from within the participating primary care and MMT centers. The staff and administrators will be 18+ years of age (and will have finished respective professional education programs). The native and/or working language of these participants will be Ukrainian or Russian. We do not anticipate participants to speak English (or to speak it fluently). These participants have high literacy (>98%) are not expected to have specific disabilities or health limitations and will generally have completed a high school education, despite being PWIDs. To ensure that all participants have a primary care provider, we will assign anyone who does not have a PCP at baseline to one at their designated clinic. This will avoid biases of patients remaining in MMT who do not have a primary care provider.

1. How will **eligibility** be determined, and by whom?

Local research assistants in each area will assess eligibility by asking about opioid dependence based on DSM-V criteria.

1. **Risks:** Describe the reasonably foreseeable risks, including risks to subject privacy, discomforts, or inconveniences associated with subjects participating in the research.

In Aim 1 and 2 we will involve human subjects, however, the data collected will be part of routine patient care in a primary care and MMT setting and the risk will be minimal.

Risks Associated with QHI data transfer: During the entirety of this study, patient information will be collected and transferred (or input directly to REDCap) to our Yale research office through REDCap and will NOT include any distinct patient identifiers. There are no risks to the patients associated with this part of the study because (1) the patients will be receiving the care they would have received based on their preference for treatment location (primary care vs. MMT site) and (2) all data will be de-identified before transfer to Yale. De-identification involves the removal of all identifying information, such as name, address, telephone number, date of birth and social security number. Age and race/ethnicity, however, will remain on the record, as it is useful for our analysis and is not individually identifying information. The de-identified data will be transferred to Yale using secure, encrypted data transfer methods using the REDCap transfer system.

Risks Associated with Qualitative Assessments: As part of the Project ECHO-IC evaluation, some clients, medical personnel and administrators from the primary care and MMT sites will participate in face-to-face interviews. Even though the participants are clients, medical professionals or administrators who will be talking about their patients, providers, or facilities, some of these topics may make some participants feel uncomfortable. Participants will be informed that they may choose not to answer all or any part of a question they are uncomfortable with during the interviews. Prior to interviewing, they will be given an information sheet and will be asked if they would like to participate. They will be given the option to take time and think about it. They may choose to withdraw from the study at any point and decide not to come in for the interviews. During the interviews, participants may choose not to answer a question or part of a question and are free to give as little or as much information as they feel comfortable. Data from the audio-taped interviews will be reviewed after they have been completed. No study-related unanticipated problems or adverse events are expected to occur in this part of the study. Hard copies of data collected will be stored in a locked cabinet behind double-locked doors. Electronic records of the interviews will be password-protected. We have successfully accomplished this strategy during our previous implementation science study in our ExMAT study in Ukraine with 199 participants in our focus groups and >60 staff members. We have also done with in our studies to integrate buprenorphine into HIV clinical care settings at 10 sites throughout the US. We have published these data and have had no untoward outcomes.[^67^](#_ENREF_67)^,^[^75^](#_ENREF_75)

Risks Associated with Staff Surveys: As part of the Project ECHO-IC evaluation, medical personnel and administrators from the participating medical centers will complete surveys. In the surveys they may be asked questions on topics that may make them feel uncomfortable. There are minimal risks expected from completing questionnaires, such as feeling uncomfortable with certain topics. Similar to the protections of the face-to-face interviews above, if anyone feels uncomfortable, they may choose not to answer any part or all of a question. During the initial consent procedures, , they will be fully informed about all parts of the questionnaires, and will be asked whether they would like to participate in the study. They may choose to withdraw from the study at any point and decide not to complete the survey but our experience has been nearly 100% acceptance since none of their individual responses will be reported and only aggregate data will be provided. No study-related unanticipated problems or adverse events are expected to occur in this part of the study. All data will be collected using Qualtrics and maintained on a Yale password protected computer.

Risks Associated with Patient Satisfaction Surveys: As part of the Project ECHO-IC evaluation, a random sample (not to exceed 10% of the patients in each facility) will be surveyed using an on-line system at each site. This will be performed using an online Qualtrics system, either on a tablet PC (or Ipad) or on a computer. There are minimal risks expected from completing questionnaires, such as feeling uncomfortable with certain topics, however, the information to be collected only relates to their satisfaction with the MMT program. The patients will be told that they can refuse to answer any of the survey if they feel uncomfortable and that their responses will be kept anonymous and will in no way affect their receipt of care or treatment at the medical facility. During initial consent procedures, they will be fully informed about all parts of the study, including the questionnaires, that they will not be identified aside from the clinic where they receive their MMT, will be asked to read the informed consent form using the online system, and will be asked whether they would like to participate in the study. They may choose to withdraw from the study at any point and decide not to complete the survey. If they choose not to complete the online survey, they will be asked why (e.g., concerned about confidentiality, too busy, etc). No study-related unanticipated problems or adverse events are expected to occur in this part of the study. Electronic copies of the questionnaires will be password-protected. In no way will their responses be relayed to the clinical staff and jeopardize the relationship with their clinical providers. Instead, it will be described that this will be used as a quality improvement project.

Risks Associated with Loss of Confidentiality: As with all research, there is a risk that involves potential breaches of confidentiality. We intend to do everything possible to reduce this risk and this is one of the process measures we propose to measure. Dr. Altice (PI) has extensive experience on the legal and ethical issues involved with conducting research studies with vulnerable populations.[^211-213^](#_ENREF_211) As part of this study, subjects who are PWID, medical practitioners or administrators will be interviewed in private rooms within their facilities. Potential sites for breaches of confidentiality include the recruitment process, the study interviews, or at data management systems. All information is stored in password-protected, encrypted computers with double-password protection for opening specified files. All confidential information (study instruments, medical records, audio files, etc.) will be recorded with study participant number only and maintained in locked cabinets within offices at the Yale AIDS Program and will only be available to be opened by the Data Manager, Study Coordinator, Co-Investigators or the Principal Investigator. Electronic databases will be maintained through password-protected computers and files and maintained at the Yale AIDS Program and the Yale Center for Analytical Sciences (YCAS).

1. **Minimizing Risks:** Describe the manner in which the above-mentioned risks will be minimized.

Clinical Expertise in ECHO-IC: To protect against risks to patients, all providers participating in ECHO-IC will receive expert guidance and training for treating these disorders in their clinical care settings. Our team of Addiction, HIV, TB and Primary Care Experts from Ukraine and US will provide expert guidance on screening and treating a number of comorbid conditions. Our clinical experts on addiction will be Dvoryak, Vivyuski, Dumchev, Haddad and Altice. Experts on HIV, HCV and TB are Atanyak, Altice, and Haddad. Experts on Primary Care are Matsuka and Haddad. Experts on Psychiatry are Dvoryak and Dumchev.

Informed Consent: Patients will be asked to provide informed consent to be randomized to either a primary care or MMT clinic. In either case they will be receiving MMT. Clients, medical and administrative personnel that will participate in in-depth interviews will be given information sheets/verbal consents by research assistants (RAs). Information sheets will be approved by the Yale IRB. Surveys will be conducted on-line using Qualtrics. All participants will be reminded that their refusal to participate will in no way negatively affect their relationship with any of the participating medical centers in the future.

Qualitative Assessments and Staff Surveys: There are minimal risks expected from participating in interviews, such as feeling uncomfortable with certain topics being discussed. As mentioned previously, all participants for interviews will be reminded that their refusal to participate will in no way negatively affect their patient-provider or professional relationship with any of the participating agencies or clinics. If participants do feel uncomfortable about certain questions asked, subjects will be able to choose not to answer the question that makes them uncomfortable. All interviews and consultations will occur in private rooms, to ensure confidentiality. All data will be stored in secure locations. Everyone will be told that the meeting and survey data are confidential.

Recruitment: Recruitment procedures are designed to reduce the risk of confidentiality loss for participants. Patients will be recruited based on their preference for treatment. Clients, medical and administrative personnel from the participating primary care and MMT centers will be invited to participate in the study and undergo interviewing and surveys. Patients will be randomly selected to complete a patient satisfaction survey. They will be reassured that their participation is voluntary and confidential.

1. **Data and Safety Monitoring Plan:** Include an appropriate Data and Safety Monitoring Plan (DSMP) based on the investigator’s risk assessment stated below. (Note: the HIC will make the final determination of the risk to subjects.) For more information, see the Instructions, page 24.

a. What is the investigator’s assessment of the overall risk level for subjects participating in this study? Minimal risk

b. If children are involved, what is the investigator’s assessment of the overall risk level for the children participating in this study? N/A

c. Include an appropriate Data and Safety Monitoring Plan. Examples of DSMPs are

available here <http://www.yale.edu/hrpp/forms-templates/biomedical.html> for

Minimal risk, but we will include a DSMB for aim 1—see below for details.

d. For multi-site studies for which the Yale PI serves as the lead investigator:

N/A; Sergii Dvoriak (UIPHP) will serve as the lead investigator in Ukraine.

- - 1. How will adverse events and unanticipated problems involving risks to subjects or others be reported, reviewed and managed?
    2. What provisions are in place for management of interim results?
    3. What will the multi-site process be for protocol modifications?

Aim 1 requires a DSMB.

Data and Safety Monitoring Board

We will comprise a full Data and Safety Monitoring Board (DSMB), that will include three voting members from colleagues in the field. The PI on this grant is familiar with the implementation of a DSMB; he is currently serving as one of the board members of the NIDA Clinical Trials Network studies examining routine HIV testing among drug users.

The key elements of a DSMB include the following:

1. Selecting experts in the field (HIV, TB, Addiction) who will serve as members of the board. If funded, we will select two U.S. and one Ukrainian experts to serve on the DSMB. As in other NIDA trials, we will work with NIDA staff to select these individuals. Individuals from NIDA will be responsible for the final invitation to participate on the Board. During the second six months of the study, after the QHI scores are selected by the physician and expert stakeholders, while final implementation is just starting, the DSMB will convene to go over their charge, examine the guidance set forth by the study team and to question the PI and biostatisticians about pre-defined endpoints. Once these outcomes and stopping rules are defined, the Board will reconvene at regularly scheduled times to assess outcomes.
2. Defining stopping rules. For Aim 1, we will be examining achievement of a composite QHI score. It is not anticipated that stopping would occur on the basis of superiority between the three arms in the trial early because of the phase-in nature of the study where there may not be enough variability in clusters (region) (5 per phase-in) to justify stopping. Because of any anticipated concern that the primary care physician may incorrectly prescribe or dispense methadone and result in harm because of not being aware of how to adequately manage more challenging patients (e.g., death from overdose or poorly managed HIV or MDR-TB), the DSMB will convene to review all adverse consequences between the arms. Death will be brought to the DSMB within 24 hours if medication related (though most deaths are related to MDR/XDR TB that is poorly treated in the public sector). In our study comparing integrated MMT and TB treatment in Ukraine, we had several deaths related to undiagnosed or inappropriately treated MDR/XDR TB. We therefore justify the use of Gene Xpert in the PC settings to ensure that diagnosed TB patients get expert treatment immediately upon diagnosis (and not wait 6-8 weeks for sputum culture results). If the death rate or discontinuation rate is 3-fold higher in any group, either in the interim analysis when 50% is enrolled or at any time within study assessment, the study will be stopped for safety purposes and a press release will be made. Tolerability issues regarding MMT treatment and clinical management of patients in the experimental arm is expected to be better due to having multiple experts on ECHO-IC sessions who will discuss case examples and provide group learning and address issues of drug interactions between MMT and HIV and TB treatments that arise from medications that induce cytochrome P450.
3. Monitoring performance of the trial: The DSMB will review any protocol violations, improper entry criteria, slow accrual or retention rates, failures of randomization, or treatment participation that may undermine the internal validity of the trial and independently make recommendations for improvement or termination should the trial be able to prove anything meaningful regardless of the modifications.
4. Assessment of possible adverse consequences of the various interventions. No specific guidance is available for DSMBs for these kinds of healthcare delivery interventions; however, we have considerable experience with integrated care and community-based treatment interventions provide a specific plan. In order to collect information about the “safety” of the intervention, one research assistant will gather safety information from three independent sources. These include the weekly ECHO-IC meetings where cases are discussed, data collected from chart review, and review of patient surveys at regular intervals (e.g., cases of discrimination by providers). These data are rich and include an impressive amount of information to guide an appropriate safety plan for this intervention. The types of information captured below are a number of items that potentially could be an adverse event for participation in this study. The findings from our study will also be reported to the DSMB for them to review and provide feedback, and if necessary, halt or refine the study. These include, but are not limited to:
   1. Breaches of confidentiality: Though we have considerable safeguards to protect against confidentiality breaches, these will be assembled and first reviewed by the PI and then to the DSMB, who might, after review provide guidance on introducing new guidance.
   2. Severe adverse events associated with increased initiation of or adherence to ART or TB medications: It is possible that the two PC groups by virtue of their integrated care have patients who initiate more (necessary) medications and thereby have more adverse side effects by virtue of the polypharmacy (to treat comorbid conditions) or even drug interactions with methadone (withdrawal). The DSMB will need to balance the benefits of treatment (justified for appropriate treatment with ART or TB medications) with the adverse side effects that occur with any medication. Moreover, we anticipate high HCV prevalence of PWIDs, therefore physicians who monitor care may identify more laboratory elevations (e.g., LFTs).
   3. Assessment of unanticipated adverse consequences: Several members of our research team, including the PI, are expert in the clinical management of PWIDs (including HIV and TB). For those on MMT who receive either ART or TB Treatment, PC doctors will be trained to monitor for symptoms of opioid withdrawal and adjust MMT dose. Alternatively, we will monitor for overdose in these subjects, diversion or drug interactions with MMT. We would also monitor for breaches of confidentiality in PT subjects. An initial investigation would be undertaken and simultaneously reported to the IRB using standard university procedures.
   4. Confidentiality of research data: Breaches of confidentiality regarding research data are more difficult to detect. For that reason, we have in place a number of safeguards including double-locked doors, locked file cabinets, confidential and dedicated fax machine and double password-protected computers. In accordance with federal and university policy, all research personnel are compliant with HIPAA training. If any breach of data confidentiality were detected, we would use similarly detailed procedures for report to the PI and IRB simultaneously.

All DSMB meetings will include an open session where the PI, coinvestigators and biostatisticians present the required (and/or additional requested information) and answer questions, followed by a closed meeting of only the DSMB members will meet and vote on each issue raised, which will be recorded by the Chair. Afterwards, an executive meeting will be convened by the board, the PI, and biostatistician will meet to hear the result of the boards and be provided a task for further investigation, if any. The DSMB Chair will put in writing the findings and disseminated if deemed appropriate.

1. **Statistical Considerations:** Describe the statistical analyses that support the study design.

Aim 1 Analytic Plan: All analyses will be according to the principle of intent-to-treat, i.e., analysis as randomized. The *primary outcome* is a composite QHI score (QHI-S), measured as a mean score at 24 months. We have powered the study based on a comparison of the means of the three groups (see below) based on Delphi-guided QHIs using HIV/MMT/TB/PC recommendations. Based on the experience of using QHIs in our other previous studies we will identify the minimum list of QHIs (see below).[^13^](#_ENREF_13)^,^[^35^](#_ENREF_35)^,^[^36^](#_ENREF_36) The final components will be decided using the Delphi method[^171^](#_ENREF_171) before study initiation by a consensus panel our Ukrainian national health and *QHI* consultants, but will minimally include HIV (monitoring for CD4, ART prescription, prophylaxis treatment, monitoring of LFTs and lipids), MMT (dosing, injection frequency, abstinence), TB (screening, latent TB prophylaxis), PC (HCV, diabetes and hypertension screening/monitoring, vaccinations, physical exams, cervical screening). The total number of QHIs achieved will be divided by the maximum for each individual x 100 to determine the QHI. Data will be assessed for missingness. A repeated measures likelihood based mixed model with missing at random (MAR) assumptions will be used for the analysis of the primary outcome to compare the three allocations, adjusted for the stratification variables (region and MMT status) plus age and gender (age and gender QHIs may vary). We will also adjust for covariates that are predictive of missingness to be consistent with the MAR assumption. Sensitivity analyses as described below will be done to assess this assumption, which is a reasonable starting point given missing data is expected to be low. Results will be displayed as point estimates with corresponding 95% confidence limits. The primary (and secondary) outcomes will be tested at the 5% (2-sided) significance level. In addition, we have planned to test for a linear trend among the treatments for the proposed outcomes, testing that P4P Integrated Care > Integrated Care with No P4P > Standard of Care. We also plan to test the treatment effects using similar methods for the MMT naïve and MMT experienced strata separately. These tests will be conducted at a Bonferroni corrected type I error level of 0.025 to control for multiple testing. The secondary outcomes will include individual QHIs for HIV, TB, MMT and PC as well as QoL and stigma. Individual QHIs will be analyzed similarly to the primary outcome. Mean stigma and QoL, including subscales, will be compared as pre/post and over time in the MMT transfer strata and will be compared using one-way ANOVA/Duncan test, with general linear models to identify differences in stigma stratified by the methadone status at the time of recruitment Mean scores will be normalized, with comparisons between the three groups, with the referent being SOC (MMT specialty setting). To address multiple testing, we will use the Benjamini-Hochberg method of controlling the false discovery rate.[^172^](#_ENREF_172)^,^[^173^](#_ENREF_173) We do not expect missing data to be a concern; we expect that we will collect more than 90% of the data at the 24 month time point. We will, however, explore all patterns of missing data and compare those with and without data to determine whether the data may be MAR or not MAR (i.e. eliminate the missing completely at random assumption). We will conduct sensitivity analyses using an appropriate missing data method, such as multiple imputation. SAS version 9.3 (Cary, NC) or higher and R will be used for all analyses. Models will be run in both software languages for reproducibility.

Power Analysis: We will need a sample size of 405 per group (total sample size of 1215), to have 90% power at p<0.05 to detect a standardized effect size of 0.10 for a null of no difference between the means of the three groups versus an alternative of at least one difference at the 24 month time point. Additionally, we will have 90% power with a 2.5% type I error rate (Bonferonni corrected) to detect a standardized effect size of 0.16 within each of the MMT strata (naïve or experienced). Conservatively, we expect a 10% loss of information at the 24-month time point. Therefore, we have inflated the sample size by 10% and will recruit a total of 1350 patients.

Aim 2 Analytic Plan

Quantitative Analyses: Analyses will focus on exploring whether different organizational factors influence achieving pre-specified QHIs. Some scales may be collinear with each other, therefore principal components will be used to determine which scales should be combined and then either treated as continuous vs ordinal (e.g., high, moderate, low) variables. Bivariate analyses—specifically organizational factors by site—will be conducted to examine trends in the influence of multi-level characteristics on the implementation and effectiveness of MMT and P4P implementation. Similarly, HIV and drug stigma may influence outcomes. We will use non-parametric χ^2^ tests and independent sample t-tests to assess stigma measures in the 3 groups over time and use (SEM AMOS.22) to perform a multi-group path analysis. To calculate indirect effects and potential mediating relationships among the variables, we will use the AMOS bootstrapping procedure[^184^](#_ENREF_184) to avoiding measurement error and underestimation of the mediation significance.[^185^](#_ENREF_185) Analyzing these trends will enable us to address important questions as: “How does organizational readiness to change influence retention of PWIDs receiving methadone in primary care?” “Are certain subgroups of patients (e.g., younger vs. older) more likely to be retained on MMT and engage in PC services?” Do changes in stigma (individual, healthcare setting) impact MMT retention or achievement of QHIs. “Does the doctor/patient relationship influence QHI outcomes? “Does PC patient caseload influence the delivery of MMT or P4P on QHIs?” Since the time period is 24 months, temporal trends for measures will also be quantitatively described by providing parameters of best fitting parametric models (linear, quadratic/polynomial, exponential) to analyze such questions as: “Do increases in provider confidence lead to greater QHI scores, including MMT retention?”

Aim 2’s analysis will involve three distinct strategies. First, we will examine the time difference in composite and individual QHI scores, with sub-analyses for MMT naïve vs transferred patients and for PC setting +/- P4P vs MMT specialty setting. To do this, we will use the following generalized mixed linear model (with a logit link): $\boldsymbol{y}_{\boldsymbol{ij}}\boldsymbol{=}\boldsymbol{\beta}_{\boldsymbol{0}}\boldsymbol{+}\boldsymbol{\beta}_{\boldsymbol{1}}\boldsymbol{TIME}_{\boldsymbol{ij}}\boldsymbol{+}\boldsymbol{\beta}_{\boldsymbol{2}}\boldsymbol{Group}_{\boldsymbol{i}}\boldsymbol{+}\boldsymbol{\beta}_{\boldsymbol{3}}\left( \boldsymbol{Group}_{\boldsymbol{i}}\boldsymbol{*}\boldsymbol{TIME}_{\boldsymbol{ij}} \right)\boldsymbol{+}\boldsymbol{\beta}_{\boldsymbol{4}}\left( \boldsymbol{X}_{\boldsymbol{ij}} \right)\boldsymbol{+}\boldsymbol{v}_{\boldsymbol{0}\boldsymbol{i}}\boldsymbol{+}\boldsymbol{\varepsilon}_{\boldsymbol{ij}}$**,** where group will vary by either study arm or by MMT status in the RCT. Our dependent variables will include: 1) a binary indicator for whether an individual patient achieved the desired composite QHI cut-off (e.g. QHI>80% or QHI-80), as previously used to measure comprehensive and holistic integrated primary care for PWID receiving buprenorphine.[^186^](#_ENREF_186) The same approach will then be used for secondary individual QHI outcomes (HIV, MMT, TB and PC), which we have done previously when integrating addiction treatment into specialty services in the US[^35^](#_ENREF_35) and Ukraine.[^13^](#_ENREF_13) 2) composite QHI score treated as a continuous variable. Any changes in national guidelines that might influence a QHI would not be scored until after at least 1 year after publication in order to allow for uptake changes by clinicians. Our covariates will include intervention site and time trends, client-level variables including demographic characteristics, addiction severity and mental health measures, as well as organizational and provider level measures. In the second part of the analysis we will use a generalized linear mixed model to compare the change in the QHI score (%) relative to the baseline between each study arm to determine if there is incremental benefit of integrating MMT in PC settings and if it is further enhanced by P4P.

In the third part of the analysis, we will examine the role of barriers and facilitators measured using the multi-level framework that includes client-, provider-, organizational-, community-level variables. First, using principal component analysis (PCA), we will identify various dimensions of clinician and organizational behavior that might influence MMT integration into PC settings through adoption and implementation of ECHO-IC at each site, degree to which QI is implemented from each site, which would summarize the variability in the data. PCA, a transformation used for data reduction, will be applied to multi-level organizational variables. Each principal component represents a different weighted average of the various organizational and client level variables, and each explains a different aspect of the variation observed in patterns of the organization and staff member. Principal components are important ways to group variables that are independent of each other by design, which addresses the issue of multicollinearity,[^187^](#_ENREF_187)^,^[^188^](#_ENREF_188) which can undermine various modeling strategies. To test the extent to which the factors measuring barriers and facilitators are associated with higher or even increasing QHI composite scores over time, we would estimate, using a generalized linear mixed model in which the outcome would be measured at the patient level (level 1), as a dichotomous variable of attaining >80 QHI score. Among the covariates in the model, we would include an additional parameter that would incorporate the random effects associated with each provider (level 2), and a random effect associated with each site (level 3), which would be modeled as a function of estimated barrier and facilitator principle components.

C.2.e. Sample Size for aim 2: In order to conduct the proposed analysis, a sample size of 1,350 patients, provides more than sufficient power to conduct the proposed analyses. The use of PCA for parsimoniously grouping variables is a reductionist strategy to explain groupings of variables with multiple co-linearity. The time-dependent analysis allows for detection of even small changes in the primary and secondary outcome, even if the primary (or secondary) outcome increases any of the study groups. Thus, as few as 200-300 participants in each group is more than sufficient to achieve sufficient power (>0.95) to detect an intervention effect, including a synergistic effect of intervention^NoP4P^ x intervention^P4P^ and even by site type (by varying degrees of integration), over time.

Aim 3 Analytic Plan:

Aim 3 involves a Cost-effectiveness analysis. Model structure: For patient-level outcomes, Markov state transition models for drug use and HIV status/progression will be based on the adjacent figure. Monte Carlo simulation will estimate QALY gains, and associated costs under 4 scenarios: 1) No MMT; 2) MMT at specialty clinics; and MMT at PC clinics 3) without or 4) with P4P. Sex, age and starting state for the simulation will be randomly sampled from the multivariate distribution approximating the current HIV+ PWID population in Ukraine (including from ExMAT data with 1612 PWIDs recruited using RDS and includes current, previous and never MMT strata).[^191-193^](#_ENREF_191) To evaluate the cost-effectiveness of integrating MMT into PC using population outcomes, the estimated HIV transmission dynamics will be expanded using a validated dynamic compartmental model for population-level HIV transmission,[^8^](#_ENREF_8)^,^[^194^](#_ENREF_194) which includes four compartments based on drug injection and MMT status, further compartmentalized based on HIV progression and co-morbid conditions. Temporal system dynamics will deploy differential equations, model population-level QALY gains, number of averted HIV infections, and the associated costs will be modeled for no MMT vs. the 3 interventions proposed in the RCT and with varying levels of MMT and ART coverage.


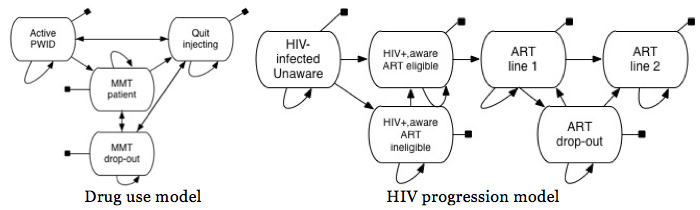


Parameters and data: State transition probabilities for the Markov model, prior distributions of rates of movement between compartments in the dynamic model, quality coefficients and costs will be estimated from currently proposed and pilot data, other ongoing studies by the PI (ExMAT), national Ukraine data and from the literature. When QALYs are the outcome, we will model the quality-adjustment coefficients for different health states as a function of QHIs and HRQoL. Transition probabilities (in the Markov model) and transition rates (in dynamic model), including mortality, will also be estimated as a function of QHIs as well as measured directly from the proposed RCT (i.e. ART initiation, ART/MMT retention, mortality, etc). Cost data will be collected from PC clinics using previously described methods.[^75^](#_ENREF_75)^,^[^195^](#_ENREF_195) The proposed modeling strategy provides an opportunity to appropriately use information about model parameter uncertainty in sensitivity and uncertainty analyses to estimate the probability of an intervention being cost-effective at different willingness-to-pay threshold levels. These empiric studies provide a unique combination of data that would allow the estimation of key model parameters, pertaining to the intervention under study, context-specific behaviors of PWIDs, and healthcare delivery in Ukraine. Parameters describing general dynamics (i.e. natural progression of HIV infection) will be estimated from literature.

Dynamic HIV transmission model calibration: A multivariate posterior model parameter distribution will be calibrated to reproduce observations from existing MoH HIV surveillance data using Bayesian Monte Carlo simulation, including plausible parameter distributions that allow the model to reproduce distinguishing features of HIV transmission dynamics in Ukraine: 1) contribution of PWIDs to new HIV infections, correcting for mis-classification;[^49^](#_ENREF_49) 2) trends for lagged HIV incidence using existing data;[^196^](#_ENREF_196) and 3) trends in HIV prevalence and incidence among PWIDs from 2009, 2011, and 2013 using RDS.[^197^](#_ENREF_197) Incremental Mixture Importance Sampling[^198^](#_ENREF_198) will be used to sample from the posterior parameter distribution, since multimodal surface of the likelihood function may create challenges for Markov Chain Monte Carlo algorithms to effectively explore the entire parameter space. One-way sensitivity analysis will be performed on all model parameters in order to identify the main parameters affecting the outcome.[^195^](#_ENREF_195) Parameter values will be varied from the lower to the upper bound of their 95% confidence intervals, or within the range of plausible values, where reliable 95% confidence intervals are unavailable. Posterior parameter distributions calibrated using Bayesian methods, will provide a basis for probabilistic uncertainty analysis, allowing Monte Carlo simulations to randomly sample from estimated posterior multivariate parameter distribution to derive the distribution of model outputs.

Uncertainty analysis: In accordance with recommended practice, we will conduct extensive sensitivity analysis to explore the robustness of our results in the face of uncertainty and to elucidate the qualitative insights arising from our quantitative analysis. We will adhere to the standards of both the US Panel on Cost-effectiveness in Health and Medicine and the International Society for Pharmacoeconomics and Outcomes Research (ISPOR) Task Force on Good Modeling Practice, adopting both deterministic and probabilistic methods of sensitivity analysis, as appropriate and feasible. We will identify instances where small input changes produce large output swings in the analysis. Each parameter in the model will be varied systematically across its 95% confidence interval in a series of “one-way” sensitivity analyses. In situations where the interplay between two or more variables may be revealing, we will use multiway sensitivity analyses and best/worst case scenarios to examine their interactive effects. We will identify “tipping point” threshold values (i.e., parameter values at which the preferred policy would change) and provide guidance for further research by constructing cost-effectiveness acceptability curves (CEACs) and evaluating the value of additional information.

## Section VI: Research Involving Drugs, Biologics, Radiotracers, Placebos and Devices

*N/A*

## Section VII: Recruitment/consent and assent procedures

1. **Targeted Enrollment: Give the number of subjects:**
   1. targeted for enrollment at Yale for this protocol___0 – all participants will be enrolled in Ukraine

b. If this is a multi-site study, give the total number of subjects targeted across all sites___ 1350 – all data will be collected in Ukraine.

1. **Indicate recruitment methods below.** Attach copies of any recruitment materials that will be used.

Flyers  Internet/Web Postings  Radio

Posters  Mass E-mail Solicitation  Telephone

Letter  Departmental/Center Website  Television

Medical Record Review  Departmental/Center Research Boards  Newspaper

Departmental/Center Newsletters  Web-Based Clinical Trial Registries

YCCI Recruitment database  Clinicaltrials.gov Registry (do not send materials to HIC)

Other (describe):

Local research assistants will recruit participants from Primary Care Centers and MMT sites.

1. **Recruitment Procedures:**
   1. Describe how potential subjects will be identified.
   2. Describe how potential subjects are contacted.
   3. Who is recruiting potential subjects?

Potential subjects will be recruited from regional areas in Ukraine through Methadone Maintenance Treatment (MMT) centers and Primary Care Centers.

Patients will be recruited based on their preference for treatment. Clients, medical and administrative personnel from the participating primary care and MMT centers will be invited to participate in the study and undergo interviewing and surveys. Patients will be randomly selected to complete a patient satisfaction survey. They will be reassured that their participation is voluntary and confidential.

1. **Screening Procedures**
   1. [Will email or telephone correspondence](file://C:\Users\mml37\AppData\Local\Microsoft\Windows\Temporary%20Internet%20Files\Content.Outlook\AppData\Local\Microsoft\Windows\Documents%20and%20Settings\jhl3\Local%20Settings\Temporary%20Internet%20Files\Content.Outlook\Local%20Settings\Temporary%20Internet%20Files\cmm82\Local%20Settings\Temporary%20Internet%20Files\jhl3\Local%20Settings\Temporary%20Internet%20Files\Local%20Settings\Temporary%20Internet%20Files\Content.Outlook\Local%20Settings\Temporary%20Internet%20Files\Content.Outlook\C0QVB04A\100%20FR%201a%20HIC%20Protocol_Application_Instructions%2006-21-10.doc#phone) be used to screen potential subjects for eligibility prior to the potential subject coming to the research office?  Yes  No
   2. If yes, identify below all health information to be collected as part of screening and check off any of the following HIPAA identifiers to be collected and retained by the research team during this screening process.

**HEALTH INFORMATION TO BE COLLECTED**:

HIPAA identifiers:

Names

All geographic subdivisions smaller than a State, including: street address, city, county, precinct, zip codes and their equivalent geocodes, except for the initial three digits of a zip code if, according to the current publicly-available data from the Bureau of the Census: (1) the geographic unit formed by combining all zip codes with the same three initial digits contains more than 20,000 people, and (2) the initial three digits of a zip code for all such geographic units containing 20,000 or fewer people is changed to 000.

Telephone numbers

Fax numbers

E-mail addresses

Social Security numbers

Medical record numbers

Health plan beneficiary numbers

Account numbers

All elements of dates (except year) for dates related to an individual, including: birth date, admission date, discharge date, date of death, all ages over 89 and all elements of dates (including year) indicative of such age, except that such ages and elements may be aggregated into a single category of age 90 or older

Certificate/license numbers

Vehicle identifiers and serial numbers, including license plate numbers

Device identifiers and serial numbers

Web Universal Resource Locators (URLs)

Internet Protocol (IP) address numbers

Biometric identifiers, including finger and voice prints

Full face photographic images and any comparable images

Any other unique identifying numbers, characteristics, or codes

1. **Assessment of Current Health Provider Relationship for HIPAA Consideration:**

Does the Investigator or any member of the research team have a direct existing clinical relationship with any potential subject?

Yes, all subjects

Yes, some of the subjects

No

If yes, describe the nature of this relationship.

1. [**Request for waiver of HIPAA authorization:**](file://C:\Users\mml37\AppData\Local\Microsoft\Windows\Temporary%20Internet%20Files\Content.Outlook\AppData\Local\Microsoft\Windows\Documents%20and%20Settings\jhl3\Local%20Settings\Temporary%20Internet%20Files\Content.Outlook\Local%20Settings\Temporary%20Internet%20Files\cmm82\Local%20Settings\Temporary%20Internet%20Files\Regulatory%20Review%20Comments%20ML.JM\100%20FR%201a%20HIC%20Protocol_Application_Instructions%2006-21-10.doc#waiver) (When requesting a waiver of HIPAA Authorization for either the entire study, or for recruitment purposes only. Note: if you are collecting PHI as part of a phone or email screen, you must request a HIPAA waiver for recruitment purposes.)

HIPAA is not applicable in Ukraine

**Choose one:**

For entire study

For recruitment purposes only

For inclusion of non-English speaking subject if short form is being used

- - 1. Describe why it would be impracticable to obtain the subject’s authorization for use/disclosure of this data;
    2. If requesting a waiver of **signed** authorization, describe why it would be impracticable to obtain the subject’s signed authorization for use/disclosure of this data;

**By signing this protocol application, the investigator assures that the protected health information for which a Waiver of Authorization has been requested will not be reused or disclosed to any person or entity other than those listed in this application, except as required by law, for authorized oversight of this research study, or as specifically approved for use in another study by an IRB.**

*Researchers are reminded that unauthorized disclosures of PHI to individuals outside of the Yale HIPAA-Covered entity must be accounted for in the “accounting for disclosures log”, by subject name, purpose, date, recipients, and a description of information provided. Logs are to be forwarded to the Deputy HIPAA Privacy Officer.*

1. **Required HIPAA Authorization:** If the research involves the creation, use or disclosure of protected health information (PHI), separate subject authorization is required under the HIPAA Privacy Rule. Indicate which of the following forms are being provided:

Compound Consent and Authorization form

HIPAA Research Authorization Form

1. **Consent Personnel:** List the names of all members of the research team who will be obtaining consent/assent.

Local research assistants from each region will be trained to consent participants. They will be required to complete Human Subjects training prior to consenting participants.

1. **Process of Consent/Assent:** Describe the setting and conditions under which consent/assent will be obtained, including parental permission or surrogate permission and the steps taken to ensure subjects’ independent decision-making.

All interviews will be done using REDCap (online and linked to study number) on an individual basis to ensure confidentiality and anonymity. For the Structured survey using REDCap, we will use strategies that we have successfully used in other surveys of medical and clinical staff (see Jin H et al and Earnshaw V et al)[^199^](#_ENREF_199)^,^[^200^](#_ENREF_200) that includes an Introduction explaining the human subjects issues involved in the survey. Each respondent will need to provide consent before advancing to the survey questions. For the face-to-face interviews, the clients, medical staff, or administrative personnel will be verbally consented and given information sheets including a description of the study, risks, benefits, privacy issues, alternatives, as well as confirmation that participation is entirely voluntary. By using the verbal consent process, we will protect the identity of the participants and no additional personal information will be collected at any point. Each interview will begin with a brief introduction including a description of the purpose of the discussion; to ensure there are no questions; and to remind interviewees that they will be audio-recorded. Interview guides will be prepared that detail the conduct during and after the interview, including not using names, not discussing specific illegal activities, and maintaining the confidentiality of participants. Patient satisfaction surveys will be conducted on-line with a subset of patients who have provided informed consent.

1. E**valuation of Subject(s) Capacity to Provide Informed Consent/Assent:** Indicate how the personnel obtaining consent will assess the potential subject’s ability and capacity to consent to the research being proposed.

The majority of the subjects will be known to the staff of the MMT or Primary Care site and if an individual has been deemed incompetent by the staff they will be excluded. If a researcher feels that the person does not comprehend what is being explained based on their interaction, consent will not be obtained. All data collection will take place in the participant’s native language, either Ukrainian or Russian to maximize comprehension.

1. **Documentation of Consent/Assent:** Specify the documents that will be used during the consent/assent process. Copies of all documents should be appended to the protocol, in the same format that they will be given to subjects.

Informed consent will be obtained from patients and medical providers prior to participating in the study. A separate verbal consent will be obtained from patients and providers that are randomly selected, and agree to participate in in-depth interviews and be audio recorded. The informed consent process will be approved by Yale and UIPHP, and completed in Ukrainian or Russian by research assistants. All patients and providers will be reminded their refusal to participate will not affect their relationship with their medical provider, or their employer, and will not affect the medical care they receive.

1. **Non-English Speaking Subjects:** Explain provisions in place to ensure comprehension for research involving non-English speaking subjects. If enrollment of these subjects is anticipated, translated copies of all consent materials must be submitted for approval prior to use.

All consent documents and survey instruments will be translated into Ukrainian or Russian depending on the primary language in the area.

**12(**a) As a limited alternative to the above requirement, will you use the short form* for consenting process if you unexpectedly encounter a non-English speaking individual interested in study participation and the translation of the long form is not possible prior to intended enrollment?

YES   NO

Note* If more than 2 study participants are enrolled using a short form translated into the same language, then the full consent form should be translated into that language for use the next time a subject speaking that language is to be enrolled.

Several translated short form templates are found on our website at:  <http://www.yale.edu/hrpp/forms-templates/biomedical.html>. If the translation of the short form is not available on our website, then the translated short form needs to be submitted to the IRB office for approval via amendment prior to enrolling the subject. ***Please review the guidance and presentation on use of the short form available on the HRPP website.***

**If using a short form without a translated HIPAA Research Authorization Form, please request a HIPAA waiver in the section above.**

1. **Consent Waiver: In certain circumstances, the HIC may grant a waiver of signed consent, or a full waiver of consent, depending on the study.** If you will request either a waiver of consent, or a waiver of signed consent for this study, complete the appropriate section below.

**Not Requesting a consent waiver**

**Requesting a waiver of signed consent**

**Requesting a full waiver of consent**

**A**. Waiver of **signed** consent: (Verbal consent from subjects will be obtained. **If PHI is collected, information in this section must match Section VII, Question 6**)

**Requesting a waiver of signed consent for Recruitment/Screening only**

If requesting a waiver of signed consent, please address the following:

a. Would the signed consent form be the only record linking the subject and the research?  Yes  No

b. Does a breach of confidentiality constitute the principal risk to subjects?

Yes  No

**OR**

c. Does the research activity pose greater than minimal risk?

Yes ***If you answered yes, stop. A waiver cannot be granted.*** Please note: Recruitment/screening is generally a minimal risk research activity

No

**AND**

d. Does the research include any activities that would require signed consent in a non-research context?  Yes  No

**Requesting a waiver of signed consent for the Entire Study (**Note that an information sheet may be required.)

If requesting a waiver of signed consent, please address the following:

a. Would the signed consent form be the only record linking the subject and the research?  Yes  No

b. Does a breach of confidentiality constitute the principal risk to subjects?

Yes  No

**OR**

c. Does the research pose greater than minimal risk?  Yes ***If you answered yes, stop. A waiver cannot be granted.***  No

**AND**

d. Does the research include any activities that would require signed consent in a non-research context?  Yes  No

**B**. **Full waiver** of consent: (No consent from subjects will be obtained for the activity.)

**Requesting a waiver of consent for Recruitment/Screening only**

a. Does the research activity pose greater than minimal risk to subjects?

Yes ***If you answered yes, stop. A waiver cannot be granted.*** Please note: Recruitment/screening is generally a minimal risk research activity

No

b. Will the waiver adversely affect subjects’ rights and welfare?  Yes  No

c. Why would the research be impracticable to conduct without the waiver?

d. Where appropriate, how will pertinent information be returned to, or shared with subjects at a later date?

**Requesting a full waiver of consent for the Entire Study (Note: If PHI is collected, information here must match Section VII, question 6.)**

If requesting a full waiver of consent, please address the following:

a. Does the research pose greater than minimal risk to subjects?

Yes ***If you answered yes, stop. A waiver cannot be granted.***

No

b. Will the waiver adversely affect subjects’ rights and welfare?  Yes  No

c. Why would the research be impracticable to conduct without the waiver?

d. Where appropriate, how will pertinent information be returned to, or shared with subjects at a later date?

## Section VIII: Protection of Research Subjects

**Confidentiality & Security of Data:**

a. What protected health information (medical information along with the HIPAA identifiers) about subjects will be collected and used for the research?

Protected health information will only be used to recruit subjects. The surveys will be confidential and only a code number will be used to identify participants.

- 1. How will the research data be collected, recorded and stored?

Assessments and surveys will be conducted using REDCap, a web-based data collection system. The assessment and survey data will have no personal information except a unique patient ID number, and will be stored on a Yale secure, password protected server. Research assistants that are responsible for recruitment and follow ups will keep in a separate REDCap portal a list of participant names, contact information, and ID numbers, only for the purposes of follow up with participants throughout the study and verifying medical record information as described in the research plan and informed consent procedures. These personal portals are only available to individual research staff, and will be kept confidential and inaccessible to other research staff or any Yale staff..

Qualitative research data will be audiotaped, during which process no identifying information will be collected. Personal details about the patient, including recruitment and screening information, will be stored on a Word document on a secure computer purchased exclusively for this research project. After audio-recording interviews, they will promptly be transcribed and stored on the secure, password protected server. The audio file will be digital; no physical tape will be kept. The transcribed files will be kept under lock and key to prevent release. No personal identifiers of participants will be recorded on the transcriptions.

c. How will the digital data be stored?  CD  DVD  Flash Drive  Portable Hard

Drive  Secured Server  Laptop Computer  Desktop Computer  Other

d. What methods and procedures will be used to safeguard the confidentiality and security of

the identifiable study data and the storage media indicated above during and after the subject’s participation in the study?

All data from participants will be collected using an on-line system and it will be de-identified upon completion of all data collection. The personal REDCap portals that stored participant names and contact information separately, for purposes of recruitment and medical record verification, will be deleted. Any data collected in the audiotaping of interviews will contain no evidence of the participant’s name or other identifying information, except for the subject’s unique code. Any hard copy data will be secured under lock and key.

Access to the data will be password protected and each research team member will have a unique ID and password to gain access.

Do all portable devices contain encryption software?  Yes  No

*If no, see* <http://hipaa.yale.edu/guidance/policy.html>

e. What will be done with the data when the research is completed? Are there plans to destroy the identifiable data? If yes, describe how, by whom and when identifiers will be destroyed. If no, describe how the data and/or identifiers will be secured.

Once data collection is completed, we will continue to store only de-identified data. All data pertaining to individual participants will be erased after the subject has discontinued participation in the study. The personal REDCap portals that stored participant names and contact information separately, for purposes of recruitment and medical record verification, will be deleted.

f. Who will have access to the protected health information (such as the research sponsor, the investigator, the research staff, all research monitors, FDA, Yale Cancer Center Data and Safety Monitoring Committee (DSMC), SSC, etc.)? (please distinguish between PHI and de-identified data)

Research staff that are responsible for recruitment and follow up will have access to patient names and contact information, for purposes of follow-up and medical record data verification during the study only. During the study, no other staff will have access to identified data. Data will subsequently be de-identified as described above.

g. If appropriate, has a [Certificate of Confidentiality](http://www.yale.edu/hrpp/resources/docs/400PR2CoC.pdf) been obtained?

h. Are any of the study procedures likely to yield information subject to mandatory reporting requirements? (e.g. HIV testing – reporting of communicable diseases; parent interview -incidents of child abuse, elderly abuse, etc.). Please verify to whom such instances will need to be reported.

## Section IX: Potential benefits

**Potential Benefits:** Identify any benefits that may be reasonably expected to result from the research, either to the subject(s) or to society at large. (Payment of subjects is not considered a benefit in this context of the risk benefit assessment.)

All PWID enrolled in the study will benefit from receiving MMT. Measures of QHI and stigma will assess the benefits provided to the clients. Medical personnel will benefit from the training obtained through Project ECHO-IC methodologies.

## Section X: Research Alternatives and Economic Considerations

1. **Alternatives:** What other alternatives are available to the study subjects outside of the research?

Subjects may receive MMT in either a Primary Care site or MMT site regardless of participation in this research.

2. **Payments for Participation (Economic Considerations):** Describe any payments that will be made to subjects, the amount and schedule of payments, and the conditions for receiving this compensation.

PWIDs enrolled as participants in the study will receive compensation for participation in the study for interviews completed every 6 months. They will be paid the equivalent of $10 USD per interview to cover their time and transportation costs as is standard in Ukraine. Staff and administrators will not be financially compensated for any of the surveys or interviews. Providers, however, will be paid for group QHI scores in the P4P portion of the study. This will come as a bonus at the end of the study based on the QI scores and will be pre-specified during the stakeholder meeting to decide a priori rules for deciding who and who will not be able to participate in the P4P incentive plan. Only the 30 physicians that are at the 15 P4P sites will be able to share the incentives, but will be based on pre-specified achievements in QHIs. **All physicians involved in the study at all of the sites will be informed of the final list of QHIs selected from the Delphi process.**

3. **Costs for Participation (Economic Considerations):** Clearly describe the subject’s costs associated with participation in the research, and the interventions or procedures of the study that will be provided at no cost to subjects.

There will be no costs to subjects for participating in this study.

4. **In Case of Injury:** This section is required for any research involving more than minimal risk.

This study involves no more than minimal risk.

a. Will medical treatment be available if research-related injury occurs?

b. Where and from whom may treatment be obtained?

c. Are there any limits to the treatment being provided?

d. Who will pay for this treatment?

e. How will the medical treatment be accessed by subjects?
